# Nanocomposites based on nanoceria regulate the immune microenvironment for the treatment of polycystic ovary syndrome

**DOI:** 10.1186/s12951-023-02182-w

**Published:** 2023-11-07

**Authors:** Sisi Yan, Zhipeng Gao, Jinli Ding, Suming Chen, Zehao Wang, Wenyi Jin, Bing Qu, Yi Zhang, Lian Yang, Duanying Guo, Tailang Yin, Yanbing Yang, Yan Zhang, Jing Yang

**Affiliations:** 1 Reproductive Medical Center, Renmin Hospital of Wuhan University and Hubei Clinic Research Center for Assisted Reproductive Technology and Embryonic Development, Wuhan, 430060 China; 2 Key Laboratory of Biomedical Polymers of Ministry of Education, College of Chemistry and Molecular Sciences, Wuhan University, Wuhan, 430072 People’s Republic of China; 3 The Institute for Advanced Studies, Wuhan University, Wuhan, China; 4 Department of General Surgery, Renmin Hospital of Wuhan University, Wuhan, China; 5 Longgang District People’s Hospital of Shenzhen, Shenzhen, China; 6 Department of Clinical Laboratory, Renmin Hospital of Wuhan University, Wuhan, Hubei China

**Keywords:** CeO_2_@RSV, PCOS, Macrophage polarization, Granulosa cells, Inflammation

## Abstract

The immune system is closely associated with the pathogenesis of polycystic ovary syndrome (PCOS). Macrophages are one of the important immune cell types in the ovarian proinflammatory microenvironment, and ameliorate the inflammatory status mainly through M2 phenotype polarization during PCOS. Current therapeutic approaches lack efficacy and immunomodulatory capacity, and a new therapeutic method is needed to prevent inflammation and alleviate PCOS. Here, octahedral nanoceria nanoparticles with powerful antioxidative ability were bonded to the anti-inflammatory drug resveratrol (CeO_2_@RSV), which demonstrates a crucial strategy that involves anti-inflammatory and antioxidative efficacy, thereby facilitating the proliferation of granulosa cells during PCOS. Notably, our nanoparticles were demonstrated to possess potent therapeutic efficacy via anti-inflammatory activities and effectively alleviated endocrine dysfunction, inflammation and ovarian injury in a dehydroepiandrosterone (DHEA)-induced PCOS mouse model. Collectively, this study revealed the tremendous potential of the newly developed nanoparticles in ameliorating the proinflammatory microenvironment and promoting the function of granulosa cells, representing the first attempt to treat PCOS by using CeO_2_@RSV nanoparticles and providing new insights in combating clinical PCOS.

**Supplementary Information:**

The online version contains supplementary material available at 10.1186/s12951-023-02182-w.

## Introduction

Polycystic ovary syndrome (PCOS) is one of the most common endocrine disorders and is characterized by polycystic ovaries, anovulation, and hyperandrogenism [1, 2]; PCOS affects approximately 20% of women of reproductive age [3, 4] and has lifelong consequences for health and wellbeing [5–7]. Although polygenes and multiple factors are involved in the pathophysiology of PCOS [8], effective biological targets and therapeutic drugs remain unsatisfactory. Thus, innovative treatment strategies to improve therapeutic efficacy are highly required.

Accumulating evidence strongly suggests that the proinflammatory microenvironment in the ovary plays significant roles in the pathogenesis of PCOS [9]. Macrophages, the most abundant immune cell type in the ovaries, are reported to exert considerable effects on ovarian homeostasis and function [10–12]. Traditionally, macrophages are categorized into classically activated (M1) and alternatively activated (M2) subtypes, and the balance of M1 and M2 macrophages is essential for the immunological milieu within the ovary [13]. The abundance of macrophages with the proinflammatory M1 phenotype accelerates the inflammatory response in the ovaries [14], and potentially contributes to the progression of PCOS [15, 16]. Researches have demonstrated that an imbalance in the ovarian immune status further impairs granulosa cell (GC) proliferation, ovarian follicular development and ovulation [14], resulting in poor oocyte quality and IVF outcomes [17]. Considering that macrophages act as drivers and regulators of ovarian functions [18], achievements that either reduce the amount and function of M1 or increase M2 activity are essential for the treatment of PCOS. Intriguingly, macrophages have been reported to be highly heterogeneous and can shift from one phenotype to another in response to the surrounding microenvironment [19], and studies have also confirmed that regulation of macrophage infiltration lowers the expression of proinflammatory cytokines and remarkably ameliorates the function of GCs and PCOS-associated clinical outcomes [20, 21]. Therefore, discouraging the immune activation of macrophages would be considered a promising and significant therapeutic strategy to relieve the symptoms of PCOS.

Recently, nanoceria (CeO_2_) has been widely applied in disease diagnosis and therapy due to its favorable antioxidant capacity [22]. Studies have demonstrated that CeO_2_ not only reduces reactive oxygen species (ROS) production but also suppresses the release of inflammatory cytokines by inhibiting M1 macrophage polarization [23, 24]. These results indicated the outstanding potential of CeO_2_ in scavenging excessive ROS and reducing macrophage infiltration. Resveratrol (RSV), a natural polyphenol, acts as an anti-inflammatory and antioxidant protective agent during PCOS [25, 26]. As a notable metabolic modulator, RSV can reduce the secretion of inflammatory cytokines and promote the transformation of macrophages into the M2 type [27, 28]. However, the therapeutic application of RSV remains limited because of its instability and poor systemic bioavailability [29, 30].

**Scheme 1:**
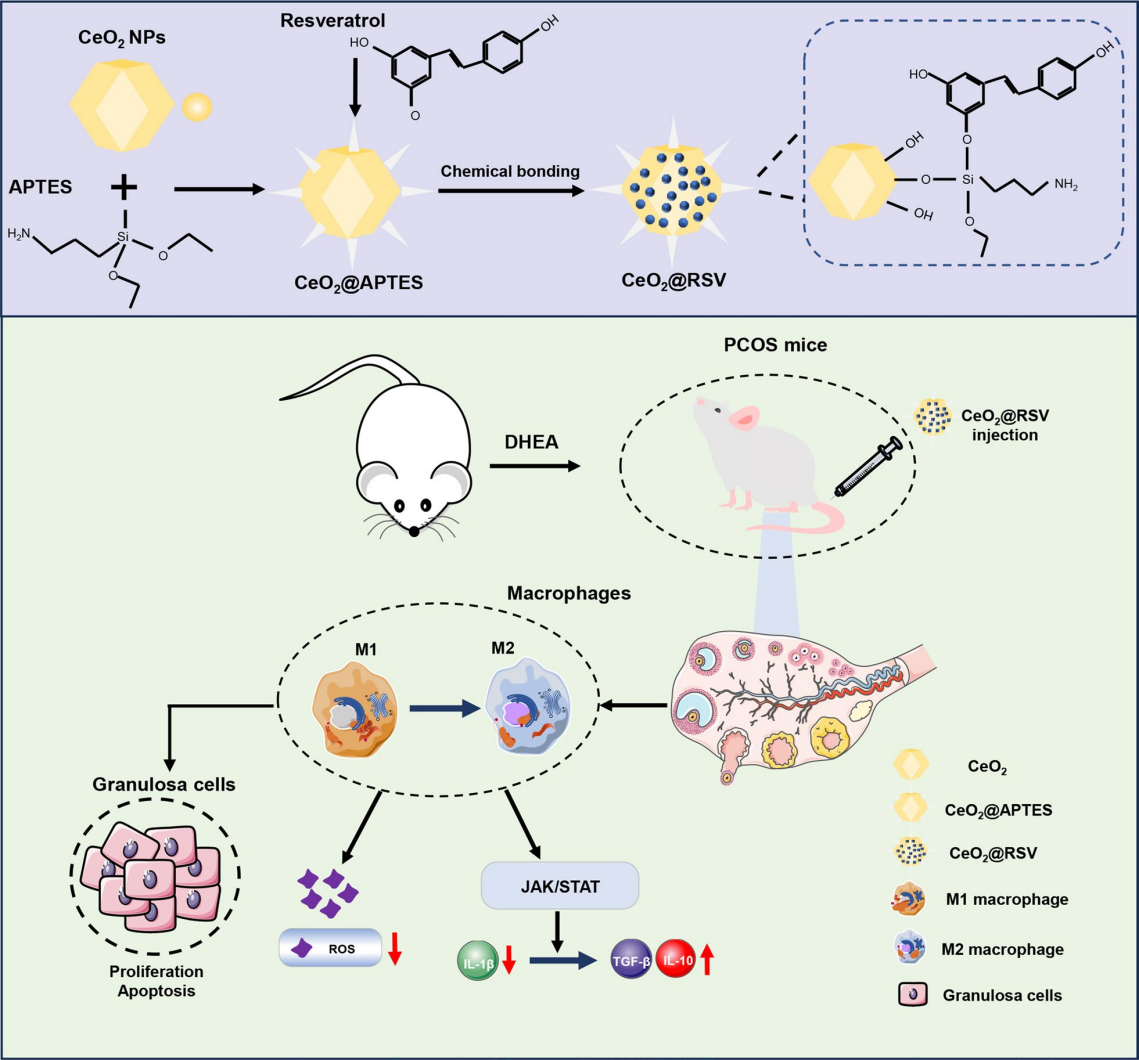
Schematic illustration of the synthesis process and anti-inflammatory functions of CeO_2_@RSV NPs

To address these issues, in the present study, we encapsulated the powerful immunomodulatory drug RSV into CeO_2_ through chemical bonding (CeO_2_@RSV), which was delivered to the ovaries to regulate the immune microenvironment and relieve the inflammatory response in PCOS (Scheme 1). The synthesized CeO_2_@RSV nanoparticles (NPs) mainly possess the following advantages: (1) The chemical bonding of RSV to CeO_2_ expands the antioxidant activity of the CeO_2_ nanozymes. (2) Surface-bound RSV may extend the anti-inflammatory capacity and play a congenerous role in dual-directional immunoregulation via macrophage polarization. (3) The CeO_2_@RSV nanocomposites also exhibited high biocompatibility and drug delivery capabilities to the ovaries and ameliorated ovarian injury in vivo. As a result, our NPs can effectively suppress inflammation response-mediated injury and alleviate endocrine dysfunction in PCOS mice, thereby achieving prominent treatment in PCOS therapy. Our findings provide new insights into the immunomodulation of PCOS and broaden the application of nanomaterials as delivery systems.

## Materials and methods

### Synthesis of the nanocomposite

#### Preparation of CeO_2_@RSV

CeO_2_ was prepared by a hydrothermal process, which was performed as previously described [31]. In brief, 0.1 mol Ce(NO_3_)_3_·6H_2_O (Siga, USA) and 0.01 mol Na_3_PO_4_·12H_2_O were dissolved in 10 mL and 30 mL of deionized water, respectively. Then, the two solutions were mixed in a Teflon container under a magnetic stirrer for 1 h and subsequently reacted for 12 h at 170 °C in a temperature-controlled electric oven (DGG-9070BD, Shenxin, Shanghai, China). The above products were washed and then dried at 50 °C in an oven.

Afterward, 3-aminopropyltriethoxysilane (APTES) was applied to prepare amino-functionalized CeO_2_ [32]. One milliliter of APTES was added to the aforementioned prepared 40 mg nanoparticles dispersed with 20 mL of absolute isopropyl ethanol and stirred vigorously for 6 h at 85 °C. A schematic image of the chemical bonding between RSV and APTES is depicted in Additional file 1: Fig. S1. Subsequently, different concentrations of RSV (5, 10, and 15 mg) were added to the amino-functionalized CeO_2_@APTES (40 mg) and dispersed into 20 mL ethanol, followed by mixing for 24 h. The final CeO_2_@RSV product was centrifuged at 9500 rpm for 15 min and dried at 50 °C in an oven overnight.

#### Characterization of synthesized nanocomposites

The morphology and structure of the NPs were characterized using transmission electron microscopy (TEM, H-8100IV, Hitachi, Tokyo, Japan) and scanning electron microscopy (SEM, JSM-IT300, JEOL, Tokyo, Japan). XRD (Empyrean, PANalytical B.V. Netherlands) was used to examine the crystalline structure of the nanoparticles by comparison with the standard XRD profile of CeO_2_, and the contents of Ce^4+^ and Ce^3+^ were determined by XPS (Escalab 250Xi, Thermo Fisher Scientific). The zeta potential was determined using a zeta potential instrument (Zetasizer, Nano-Z, Malvern Instruments Limited, UK). AIR-FTIR (VERTEX 70, Bruker, Germany) was used to measure functionalized CeO_2_ with a Bruker Vertex 80 V spectrometer. The size distribution of the nanoparticles was determined by DLS (Zetasizer Nano-ZS ZEN3700, Malvern Instruments Limited, UK). An Avance III HD 400 MHz NMR spectrometer (Bruker-BioSpin, Rheinstetten, Germany) was used to confirm the chemical bonding between RSV and APTES.

#### Drug loading efficiency (LE) and encapsulation efficiency (EE)

To determine the drug loading and encapsulation efficiency, the prepared drug-loaded NPs were analyzed by spectrophotometry at a wavelength of 372 nm. The drug loading and encapsulation efficiency were calculated using the following equations.

$${\text{Drug}}\;{\text{loading}}\;{\text{efficiency}}\left( \% \right) = \left( {{\text{total}}\;{\text{drug}}-{\text{free}}\;{\text{drug}}} \right)/{\text{weight}}\;{\text{of}}\;{\text{nanocomposites}} \times 100\% ,$$

$${\text{Encapsulation}}\;{\text{efficiency}}\left( \% \right) = \left( {{\text{total}}\;{\text{drug}}-{\text{free}}\;{\text{drug}}} \right)/{\text{total}}\;{\text{drug}} \times 100\% .$$

### Patients and tissue samples

The study was approved by the Ethics Committee of the Renmin Hospital of Wuhan University (Ethical Approval Number WDRY 2019-K077), and written informed consent was obtained from each participant. Human primary ovarian granulosa cells (GCs) were collected from patients at the Reproductive Center of Renmin Hospital of Wuhan University. PCOS patients (n = 10) were diagnosed according to the 2003 Rotterdam Criteria [33]. The baseline characteristics of the patients are described in Additional file 1: Table S1.

GCs were obtained from PCOS patients who had undergone oocyte retrieval with a long stimulation protocol and isolated by density gradient centrifugation. Briefly, the follicular fluid was centrifuged at 300×*g* for 10 min, and the supernatant was discarded. The pellet was suspended in PBS (Servicebio, China), 50% Percoll (Biosharp, Wuhan) was added, and the mixture was centrifuged at 1800 r/min for 20 min. The middle white granulosa cell layer was aspirated and washed with PBS, and then the cells were seeded in a cell culture plate for further experiments.

### Animal models and treatment

#### Animal models

Female C57BL/6 mice (3 weeks) received adaptive feeding for 1 week. The PCOS mouse model was constructed as previously described [34], and the mice were injected (s.c.) with dehydroepiandrosterone (DHEA) (6 mg/100 g/d in olive oil) daily for 21 consecutive days. This study was divided into five subgroups (n = 5/group): (1) sham group [olive oil, subcutaneous (s.c.)], (2) PCOS group, (3) CeO_2_ group [mice injected with DHEA and treated with CeO_2_ with the same amount of CeO_2_ as CeO_2_@RSV], and (4) RSV group [mice injected with DHEA and treated with RSV with the identical amount of RSV as CeO_2_@RSV]. (5) CeO_2_ + RSV group [mice injected with DHEA and treated with a mixture of RSV and CeO_2_], and (6) CeO_2_@RSV group [mice injected with DHEA and treated with CeO_2_@RSV (1 mg/kg)]. For the JAK/STAT inhibitor (WP1066) study, mice were injected with DHEA and treated with CeO_2_@RSV+WP1066. All experimental groups were injected with the corresponding solution by the tail vein every other week. Then the mice were sacrificed by isoflurane anesthesia 2 weeks after the treatment. Blood samples were collected from the eyes of mice and then centrifuged and stored at − 80 °C for biochemical analyses and ELISA. All in vivo studies were performed under the approval of Animal Care and Use Committee of the Wuhan University (Ethical Approval Number 20190710).

#### In vivo fluorescence imaging

ICG-labeled NPs were synthesized as previously described [35]. In short, 40 mg CeO_2_ NPs were added into 1 mL APTES (resuspended in absolute isopropyl ethanol solution), and an indocyanine green (ICG) solution (10 mg/mL, 1 mL) was added and stirred for 6 h. Twenty milliliters of 10 mg RSV ethanol solution was then added to the mixed solution and stirred for 24 h. ICG-labeled CeO_2_@RSV NPs (1 mg/kg) were intravenously injected into mice, and the mice were sacrificed 2 weeks after the injection. Fluorescence imaging was recorded by an IVIS Spectrum (Perkin Elmer, USA). Finally, the mice were killed, and the ovarian tissues were detected by IVIS Spectrum.

### Cytocompatibility biocompatibility assay for CeO_2_@RSV

A Cell Counting Kit-8 (CCK-8) assay was conducted to investigate the cytotoxicity of our NPs in vitro. Cells were seeded at a density of 3 × 10^3^ in a 96-well plate overnight and administered different concentrations of NPs for 24 h, 48 h and 72 h. Then, 10 µl CCK-8 reagent was added to each well and incubated at 37 °C for 1 h. Cell viability was determined by using a microplate reader (Ensight, Perkin Elmer, Waltham, MA, United States) at 450 nm. All CCK-8 tests were performed in triplicate and were repeated three times.

In vivo, healthy C57BL/6 mice (n = 5) were intravenously administered NPs (1 mg/kg) as a bolus (100 μL) through the tail vein. After 2 weeks, the primary organs (including the heart, liver, spleen, kidney, and ovary) were collected and fixed in formalin, followed by H&E staining and pathological analysis. The stained tissue samples were observed under a microscope (Olympus BX53) at a magnification of 200×.

### Cell culture and treatment

#### Cell culture

The human ovarian granulosa cell line (KGN cells) was acquired from the Institute of Biochemistry and Cell Biology, the primary ovarian granule cells (GCs) obtained from PCOS patients were grown in DMEM/F-12 medium (Gibco, China), and the human monocyte cell line THP-1 was maintained in RPMI-1640 medium (Gibco) with 10% FBS (Gibco) at 37 °C in 5% CO_2_.

#### Effect of nanocomposites on immunoregulation via macrophage polarization in vitro

Based on the cytotoxicity tests of the different concentrations of CeO_2_@RSV (10, 50, and 100 µg/mL), the concentration of 50 µg/mL NPs was suitable and used in the subsequent experiments.

THP-1 cells were incubated with phorbol 12-myristate 13-acetate (PMA, Sigma, 16561-29-8, USA) for 24 h to differentiate into M0 macrophages. To investigate the impact of NPs on the repolarization of M1 macrophages, M0 macrophages were cultured with 100 ng/mL LPS (Sigma, L4391, USA) plus 20 ng/mL IFN-γ (PeproTech, 300-02, USA) to induce the M1 phenotype. Similarly, a concentration of 50 µg/mL of prepared NPs was added to M1 macrophages according to the CCK8 results of the macrophages. After incubation for 48 h, the supernatant was centrifuged at 2000 rpm for 10 min, and the cells and supernatant were collected for further analysis. For inhibition of signaling pathways, macrophages were pretreated with 5 µM WP1066 (MCE, China), 10 µM BAY11-7082, 100 nM VX-11e or 10 µM SB 239063 for 12 h before further experiments.

#### Coculture model system

To investigate how macrophage polarization affects the proliferation and apoptosis of KGN cells in vitro. A cell coculture model system was used, wherein NP-treated M1 macrophages were added to the upper chamber of a Transwell chamber system, and KGN cells were placed in the lower chamber. Then, the cells were evaluated after coculturing for 48 h.

### Flow cytometry

Fresh mouse spleens were acquired and ground and then filtered with a strainer, and a red blood cell lysis solution (Servicebio, China) was added to lyse the red blood cells. Ground spleen-derived cells or GC cells in different treatment groups were separated in 200 μL of transcription factor buffer (BD Pharmingen), and the corresponding antibodies were incubated at 4 °C for 30 min. For apoptosis analysis, an Annexin V-FITC/PI Apoptosis Detection Kit (ABclonal, China) was used to stain cells at room temperature for 15 min in the dark. Next, the cells were washed with PBS, and flow cytometry analysis was performed using a Beckman CytoFLEX flow cytometer. The data were analyzed with Flow Jo software (Tree Star, Inc., Ashland, OR USA).

### Histological assessment 

Tissues were fixated in 4% formaldehyde for 24 h, dehydrated and embedded in paraffin. Then, the sections were cut at a thickness of 5  μm for hematoxylin and eosin (H & E) stain. The sections were stained with hematoxylin and dehydrated by graduated ethanol, and the sections were sealed with neutral resin. Representative tissue sections were imaged under a light microscope (Nikon, Tokyo, Japan).

### Immunofluorescence

After fixation with 4% formaldehyde, the cells were incubated with primary antibodies against iNOS or CD206 (Affinity, Cat. No. DF4149 and AF0199, dilution 1:500) at 4 °C overnight. The corresponding secondary antibodies (1:200) were applied for 2 h, and the nuclei were stained with 4′,6-diamidino-2-phenylindole (DAPI, Servicebio, China). The results were observed with a fluorescence microscope (Olympus, Tokyo, Japan).

### Real-time polymerase chain reaction (RT-PCR)

Total RNA was extracted using TRIzol reagent (Accurate Biology, China) according to the manufacturer’s instructions. Reverse transcription was conducted with the PrimeScript RT reagent kit (Accurate). PCR was performed by using a 7500 Real-Time PCR system (Applied Biosystems, Foster City, CA, USA). The relative gene expression levels reported in this study were analyzed with the 2^−ΔΔ^ Ct method. The primers used to measure mRNA expression levels are shown in Additional file 1: Table S2.

### Western blot analysis

Proteins were extracted from tissues and cells with RIPA lysis buffer containing protease inhibitors. Proteins were separated by SDS-PAGE and transferred onto a PVDF membrane (EMD Millipore, Bedford, MA, USA). The membranes were blocked in 5% BSA, and subsequently primary antibodies against actin (ABclonal, Cat. No. A17910), Bcl2, Bax (Proteintech, Cat. No. 68103-1-Ig, 60267-1-Ig), STAT3 (Proteintech, China, No. 10253-2-AP), p-STAT3 (Affinity, China, Cat. No. AF3293), NF-κB, p-NF-κB (Cell Signaling, USA, Cat No. 8242, 3303), Erk1/2, p-Erk1/2 (Cell Signaling, Cat No. 4696, 4376), p38 MAPK, and p-MAPK (Cell Signaling, Cat No. 8690, 4511) were incubated overnight at 4 °C. HRP-conjugated secondary antibodies (Abmart, Cat. No. T55756F) were utilized, and the membranes were visualized using a chemiluminescence western detection system (Bio-Rad, Hercules, CA, USA) and analyzed by ImageJ.

### Measurement of serum biochemical markers

After collecting the supernatants of the mice, the levels of different biochemical markers [testosterone (T), estradiol (E_2_), luteinizing hormone (LH), IL-1β and IL-10] were measured by sandwich ELISA (R&D Systems) according to the manufacturer’s instructions. The oral glucose tolerance test (OGTT) was measured by tail vein blood sampling using a blood glucose meter (ONETOUCH Ultra Vue, China). Blood glucose levels were measured after fasting and then administered glucose (2 g/kg body weight). The glucose uptake ability and the level of lactic acid were evaluated by using a lactic acid determination kit (A019-2-1, Nanjing Jiancheng) and glucose determination kit (A154-1-1, Nanjing Jiancheng) according to the manufacturer’s instructions.

### Malondialdehyde (MDA), catalase (CAT) and superoxide dismutase (SOD) tests

MDA (A003-1, Jiancheng, Nanjing), CAT (S0051, Beyotime) and SOD (A001-3, Jiancheng, Nanjing) assay kits were used to assess the levels of MDA, CAT and SOD. Then, the corresponding solutions were added to the working solution following the manufacturer’s instructions.

### ABTS free radical scavenging assay

An ABTS scavenging assay was used to evaluate the antioxidant activity of NPs according to the corresponding instructions (SO121, Beyotime). The different NP samples (10 μL) were mixed with peroxidase working solution (20 μL) + ABTS working fluid (170 μL) and then examined at 734 nm absorbance.

### Measurement of intracellular ROS levels

The cellular ROS levels were evaluated with 2′,7′-dichlorofluorescein diacetate (DCFH-DA) as previously described [36]. Briefly, the treated KGN cells were suspended in 1 mL of serum-free medium supplemented with 1 μL of the oxidative fluorescent dye DCFH-DA (S0033, Beyotime) for 30 min at 37 °C. Finally, the intensity of the DCFDA signal in the cells was detected by flow cytometry.

### EdU and terminal deoxynucleotidyl transferase-mediated dUTP nick-end labeling (TUNEL) assay

The proliferation of KGN cells was assessed using an EdU cell proliferation kit (C0078S, Beyotime, China). After treatment, serum-free DMEM/F12 medium containing 1:1000 EdU was incubated for 2 h. The apoptosis of ovarian tissues was assessed using a TUNEL apoptosis assay kit (C1086, Beyotime, China). The cells and ovarian paraffin sections were washed with PBS, fixed in 4% paraformaldehyde for 1 h and permeabilized with 0.1% Triton X-100 for 5 min. Then, the cells and paraffin sections were incubated with the TUNEL working solution in the dark for 30 min at 37 °C. Three randomly selected fields in each sample were observed, and the percentage of positive cells was calculated.

### RNA sequencing

RNA sequencing was performed to investigate the underlying mechanisms of the anti-inflammatory effect of CeO_2_@RSV. Briefly, PMA-induced M0 macrophages were seeded in 6 cm plates at a density of 5 × 10^6^ cells/well, and then 100 ng/mL LPS plus 20 ng/mL IFN-γ and 50 μg/mL CeO_2_@RSV were cultured for 48 h. TRIzol reagent was used to collect the total RNA, and the gene expression level was analyzed by Wekemo Technologies (Shenzhen, China). Gene Ontology (GO) enrichment analysis and Kyoto Encyclopedia of Genes and Genomes (KEGG) pathway enrichment analysis were performed using Wekemo BioinCloud (https://bioincloud.tech/).

### Statistics

All statistical analyses were conducted using GraphPad Prism 6 software. The results are presented as the mean ± SD. Paired t tests were used when only two groups were compared. Evaluation of significance was performed using one-way ANOVA when more than two groups were compared. *P* < 0.05 was used to indicate statistical significance.

## Results

### Characteristics of prepared NPs

**Fig. 1:**
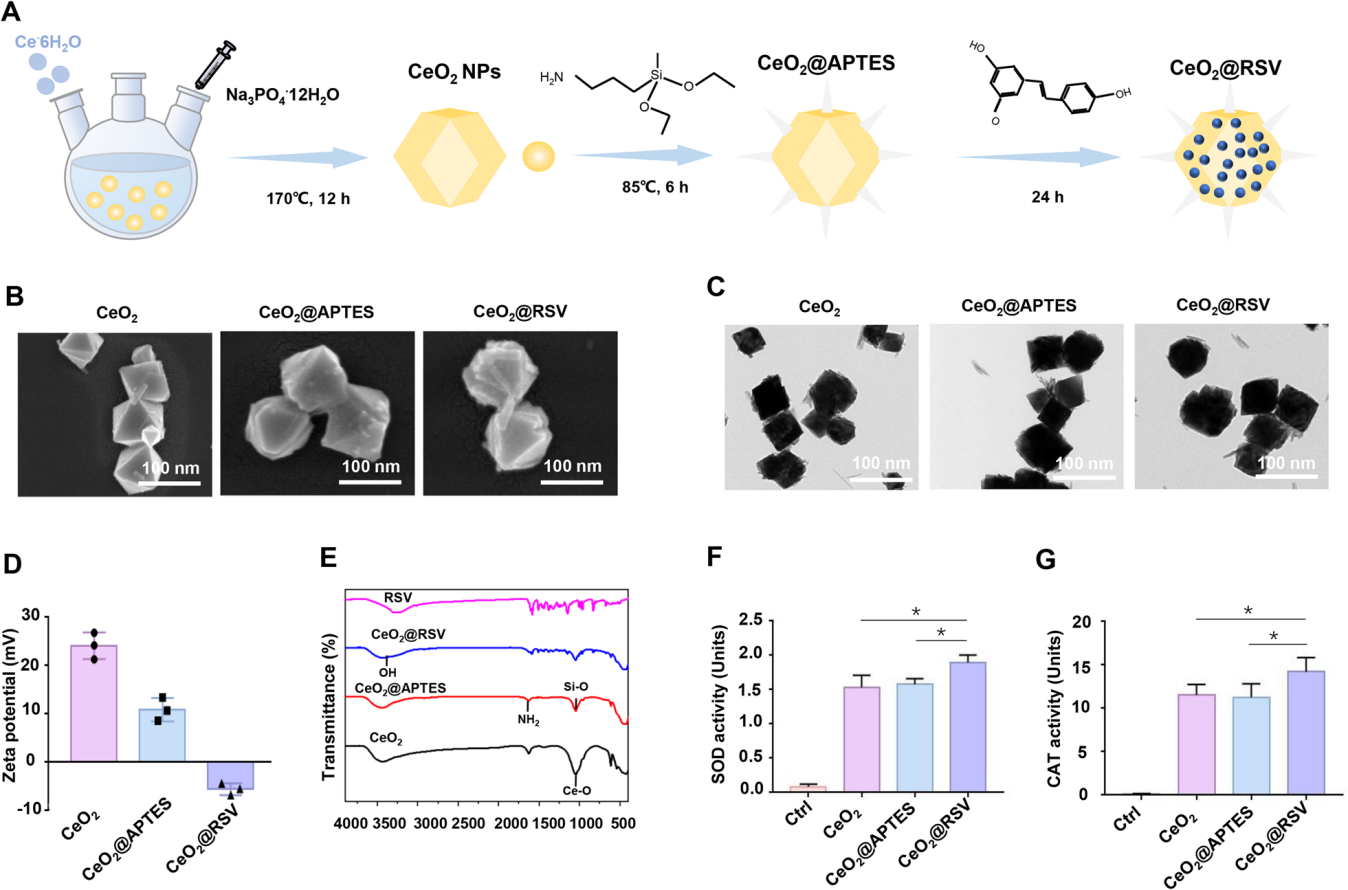
Characteristics of prepared NPs. **A** Schematic representation of the synthesis of CeO_2_@RSV. **B** SEM images of CeO_2_, CeO_2_@APTES and CeO_2_@RSV. **C** TEM images, **D** zeta potential, **E** FTIR, **F** SOD activity and **G** CAT activity of the indicated NPs. Data are shown as the mean ± SD. n = 3, *P < 0.05. Scale bar: 100 nm

Octahedral CeO_2_ was functionalized with 3-aminopropyltriethoxysilane (APTES) to form Si–O bonds, followed by chemical bonding of RSV to CeO_2_ through acidic hydroxyl groups of resveratrol replacing the ethoxy groups provided by APTES, finally obtaining CeO_2_@RSV NPs. A schematic of the chemical bonding between RSV and APTES is shown in Fig. 1A. Different concentrations of RSV (5, 10, and 15 mg) were attached to CeO_2_@APTES (40 mg) by mixing for 24 h. According to the LE and EE results of the synthesized CeO_2_@RSV NPs, we found that 10 mg RSV obtained appropriate LE and EE levels and was selected for subsequent research (Additional file 1: Fig. S2, Table S3). All NPs were octahedral in shape with a uniform particle size of approximately 100 nm, as determined by TEM and SEM (Fig. 1B, C), and the average sizes of CeO_2_, CeO_2_@APTES and CeO_2_@RSV were 93, 105 and 120 nm, respectively (Additional file 1: Fig. S3). The zeta potentials of the three kinds of NPs changed from + 24.1 to − 5.6 mV, which was due to the modification of RSV, as RSV has a negative potential. (Fig. 1D). The formation of the synthesized particles was further assessed by FTIR spectrometry. As shown in Fig. 1E, a peak at 1573 cm^−1^ was associated with the NH_2_ group, which was attributed to the presence of APTES. The characteristic peak of stretching vibration at approximately 1660 cm^−1^ was found in CeO_2_@RSV, indicating the chemical bond between CeO_2_ NPs and RSV. The corresponding EDX mapping images displayed the distribution of Ce, Si and C in the nanocomposites (Additional file 1: Fig. S4). As shown in the X-ray diffraction (XRD) patterns, the characteristic peaks of the three NPs could be indexed to the ceria-based crystallographic structure (Additional file 1: Fig. S5). These results demonstrated the successful functionalization of CeO_2_ with APTES and RSV.

Given that the electron transfer between Ce^3+^/Ce^4+^ determines the nanozyme activity of CeO_2_ [37, 38], we then examined the proportion of Ce^3+^ and Ce^4+^ in the three NPs by X-ray photoelectron spectroscopy (XPS) (Additional file 1: Fig. S6). The ratios of Ce^4+^/Ce^3+^ in the three samples were 16.6%, 17.8% and 18.4%, respectively, and slight changes in the Ce^3+^ concentration indicated the stable antioxidant activity of the nanoceria when combined with RSV. The SOD and CAT activity assays demonstrated that all NPs exhibited favorable antioxidant capacities, among which CeO_2_@RSV had the greatest antioxidant ability (Fig. 1F, G). Furthermore, other probes (2,2′-azinobis-(3-ethylbenzthiazoline-6-sulphonate (ABTS)), which are also commonly used in investigating antioxidant properties, were used [39], and Additional file 1: Fig. S7 shows similar antioxidant efficiency of CeO_2_@RSV. Spectrophotometer analysis indicated that the drug loading and encapsulation efficiencies were 6.13% and 67.92%, respectively. Furthermore, the diameter and zeta potential exhibited minor fluctuations within 7 days (Additional file 1: Fig. S8A, B), which confirmed the favorable stability of CeO_2_@RSV under physiological conditions.

### CeO_2_@RSV manipulates the infiltration of macrophages and reduces the inflammatory response

**Fig. 2:**
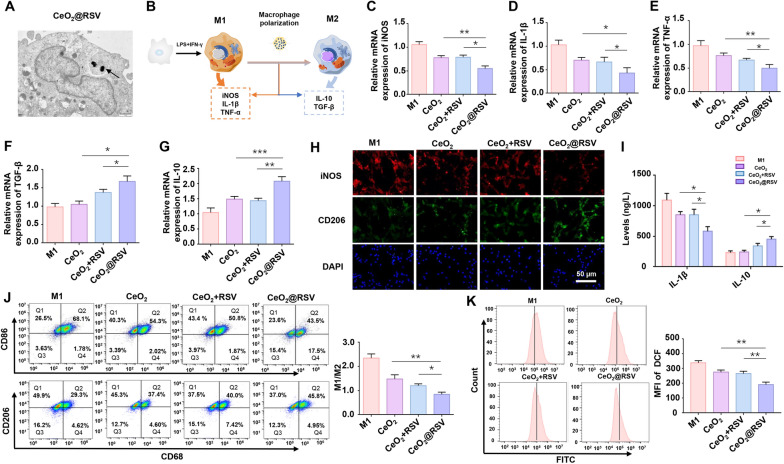
CeO_2_@RSV manipulates the infiltration of macrophages and reduces the inflammatory response. **A** Representative TEM images of macrophages incubated with CeO2@RSV (50 μg/mL) for 48 h. Scale bar: 10 µm. The arrowhead indicates intracellular NPs. **B** The diagram describes the effect of CeO_2_@RSV on the repolarization of macrophages from M1 to M2. mRNA expression levels of iNOS (**C**), IL-1β (**D**) and TNF-α (**E**) were determined by RT-PCR in M1 macrophages treated with NPs. mRNA expression levels of TGF-β (**F**) and IL-10 (**G**) in different experimental treatment groups. **H** Immunofluorescence results of iNOS and CD206 in different experimental treatment groups. Scale bar: 50 μm. **I** ELISA results of IL-1β and IL-10 levels in the supernatants of the different treatment groups. **J** Flow cytometry analysis of specific markers of M1 (CD86) and M2 (CD206) macrophages in different experimental treatment groups. **K** Representative histograms and MFIs of DCF fluorescence in M1 macrophages treated with NPs. n = 3, *P < 0.05, **P < 0.01, ***P < 0.001

Macrophage infiltration and macrophage-derived products are the main pathological factors in PCOS [14, 40]. Before applying our constructed CeO_2_@RSV NPs, we first optimized the concentrations in vitro. The cytocompatibility of CeO_2_@RSV was detected by a CCK-8 assay to assess the viability of the macrophages (Additional file 1: Fig. S9). We found that a dose of 50 µg/mL resulted in relatively high cell viability and served as the optimal concentration for subsequent experiments. In addition, TEM revealed that CeO_2_@RSV was present in the cytoplasm of macrophages, suggesting the favorable uptake and biological activity of our synthesized NPs (Fig. 2A).

We next investigated the immunoregulatory effect of CeO_2_@RSV on macrophage polarization. PMA-induced M0 macrophages were incubated with CeO_2_@RSV, and the results showed that CeO_2_@RSV markedly promoted the differentiation of M0 macrophages into M2 cells while decreasing their differentiation into M1 cells (Additional file 1: Fig. S10). In addition, LPS + IFN-γ was added to M0 macrophages prior to NPs treatment (Fig. 2B). The mRNA expression levels of M1 polarization markers (IL1-β, iNOS and TNF-α) were decreased compared with those in the control group, with the CeO_2_@RSV group exhibiting the most prominent effects (Fig. 2C–E), which indicated superior abilities owing to the synergistic effect of CeO_2_ and RSV. TGF-β and IL-10, indicating M2 markers, were upregulated in the CeO_2_@RSV group (Fig. 2F, G). Immunofluorescence also indicated increasingly high levels of CD206 but diminished levels of iNOS in the CeO_2_@RSV group (Fig. 2H). Furthermore, the level of proinflammatory IL1-β was significantly suppressed in NPs-treated cells, and the level of anti-inflammatory IL-10 increased most efficiently in the supernatant of CeO_2_@RSV-treated cells (Fig. 2I). The flow cytometry results indicated the same trend (Fig. 2J). The above results demonstrated that the CeO_2_@RSV we prepared could manipulate the M2 phenotype polarization of undifferentiated (M0) and differentiated (M1) macrophages and promote the anti-inflammatory effect of M2 macrophages; these changes in macrophage profiles are critical for remodeling the ovarian immune microenvironment.

It is known that macrophages produce excessive ROS when activated by diverse inflammatory stimuli [41], and oxidative stress may further exacerbate inflammatory reactions and arouse a vicious cycle [42]. In addition, CeO_2_@RSV NPs presented predominant SOD and CAT activity; therefore, we evaluated the effect of NPs on ROS production in inflammatory macrophages. As indicated in Fig. 2K, macrophages pretreated with NPs exhibited distinctly reduced intracellular ROS levels, with minimum DHE fluorescence. Moreover, MDA and SOD, well-established markers of oxidative stress, were measured to assess antioxidative capacity. As expected, compared to all other groups, the CeO_2_@RSV group exhibited markedly increased SOD levels and decreased MDA levels (Additional file 1: Fig. S11A, B). Collectively, these data suggest that CeO_2_@RSV NPs inhibit oxidative stress and increase the antioxidative ability of M1 macrophages.

### CeO_2_@RSV-induced macrophage polarization promotes proliferation and inhibits apoptosis in granulosa cells

**Fig. 3:**
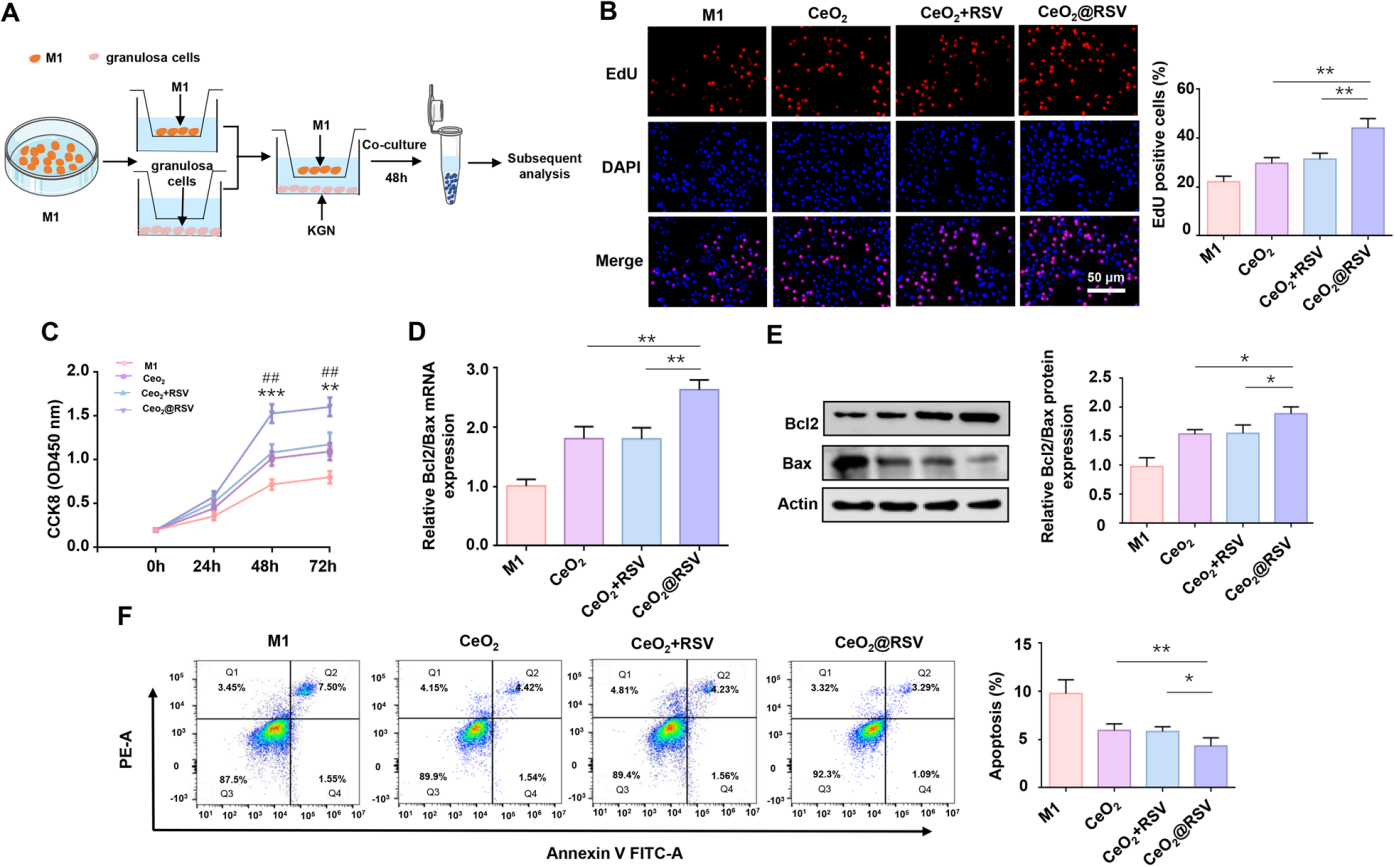
CeO_2_@RSV-induced macrophage polarization promotes proliferation and inhibits apoptosis in KGN cells. **A** Schema of the NPs-treated macrophage-KGN cell coculture model. **B** Representative images and quantification of EdU (+) in KGN cells. Scale bar = 50 μm. *P < 0.05, **P < 0.01. **C** CCK8 results of KGN cells in different groups. *P < 0.05, **P < 0.01, ***P < 0.001 vs. the CeO_2_ group; ^#^*P* < 0.05, ^##^*P* < 0.01 vs. the CeO_2_ + RSV group. **D** RT-PCR analysis of Bcl2/Bax in different groups. **E** Western blot analysis of Bcl2/Bax in different groups. **F** Apoptosis of KGN cells in each group was detected by flow cytometry. n = 3, *P < 0.05, **P < 0.01, ***P < 0.001

The proliferation and apoptosis of GCs are thought to play essential roles in the pathogenesis of PCOS [43]. Recently, much attention has been given to the effect of macrophage polarization on the abilities of GCs, and the increased M1/M2 ratio suppresses GC proliferation in antral follicles [16]. Herein, granulosa cells and NPs-treated M1 macrophages were cocultured in a noncontact Transwell system to explore the effect of NPs-treated macrophage polarization on the proliferation and apoptosis of KGN cells (Fig. 3A). The results of CCK-8 and EdU assays illustrated that the proliferation of KGN cells in the NPs-treated coculture group was augmented compared with that in the group in which KGN cells were cultured with M1 macrophages alone (Fig. 3B, C). In the apoptotic pathway, the proapoptotic molecule Bax and the antiapoptotic molecule Bcl2 are the most important participants that effectively regulate the cell [44]. Hence, to corroborate the effects of NPs-treated M1 macrophages on KGN cells, we tested the changes in the levels of these genes. As expected, compared to all other groups, the CeO_2_@RSV coculture group exhibited a markedly increased Bcl2/Bax ratio (Fig. 3D, E). Furthermore, the apoptosis of KGN cells in different groups was assessed using flow cytometry, and our results suggested that the apoptosis rate of KGN cells was dramatically decreased in the NPs-treated cocultured group compared with the M1 macrophage cocultured group (Fig. 3F). Comparatively, CeO_2_@RSV yielded a more efficient effect on the proliferation and apoptosis of KGN cells than CeO_2_ + RSV and CeO_2_ alone. Furthermore, we observed analogous phenomena in human primary ovarian GCs pretreated with CeO_2_@RSV, such as a higher Bcl2/Bax ratio and a lower apoptosis rate (Additional file 1: Fig. S12).

### CeO_2_@RSV directly changes the inflammation status and affects the apoptosis of ovarian tissues in PCOS mice

**Fig. 4:**
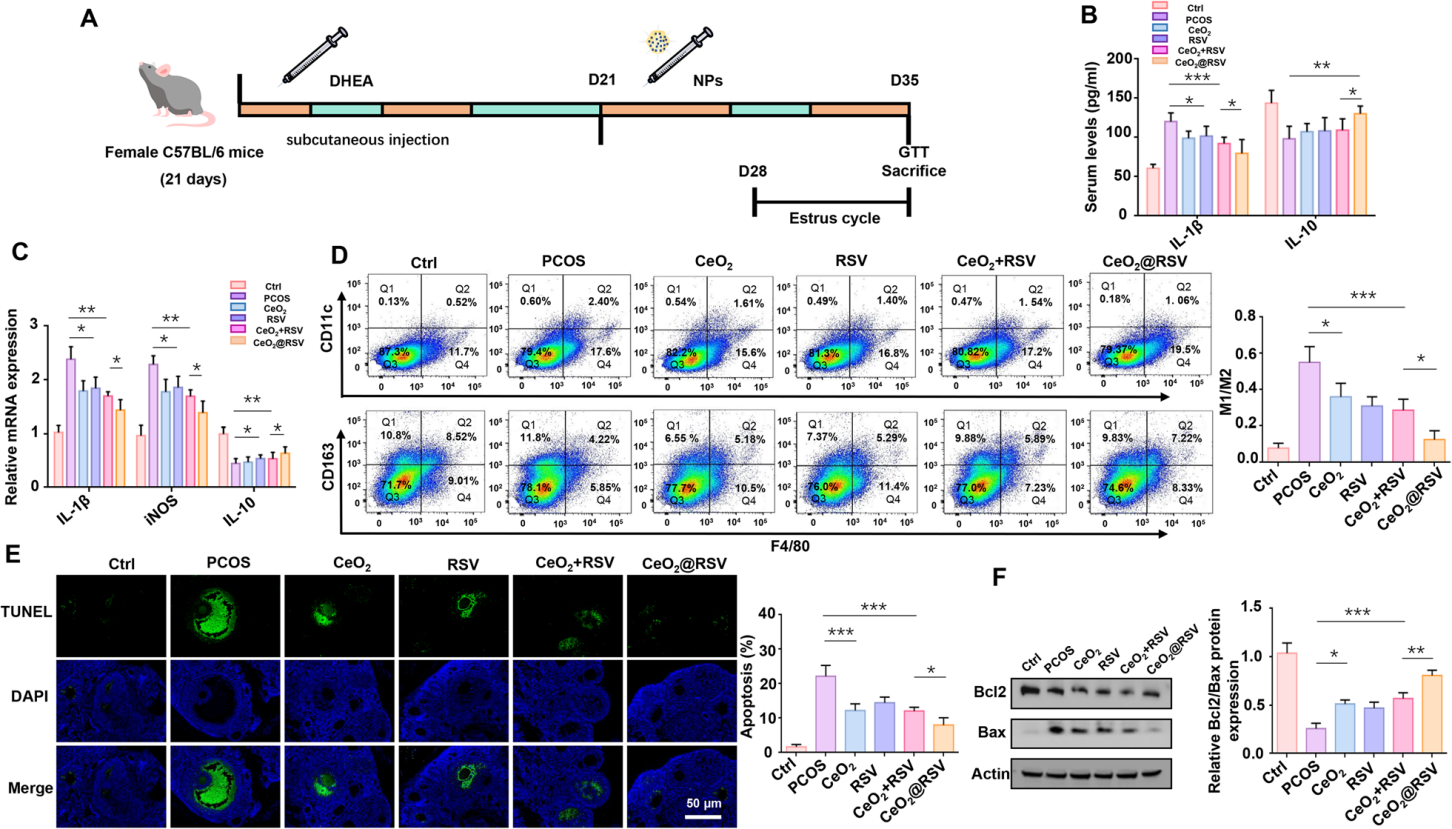
CeO_2_@RSV directly changes the inflammation status and affects the apoptosis of ovarian tissues in PCOS mice. **A** Experimental protocol for the therapeutic treatment of PCOS in mice. **B** ELISA results of IL1-β and IL-10 levels in the serum of each group (n = 5). **C** RT-PCR analysis of the mRNA expression levels of IL1-β, iNOS and IL-10 in the ovarian tissues of each group. **D** The ratio of M1 (F4/80^+^CD11c^+^)/M2 (F4/80^+^CD163^+^) macrophages in the spleens of mice from the indicated groups was analyzed by flow cytometry. **E** Representative images of TUNEL assay results in mouse ovaries from the indicated groups. Scale bar: 50 µm. **F** Western blot analysis of the Bcl2/Bax ratio in the ovarian tissues of each group. *P < 0.05, **P < 0.01, ***P < 0.001

Based on the excellent anti-inflammatory and antioxidant effects of CeO_2_@RSV in vitro, we further established a DHEA-induced PCOS mouse model and then administered CeO_2_, RSV, CeO_2_ + RSV and CeO_2_@RSV for the next 2 weeks to investigate the effects in vivo (Fig. 4A). First, an animal long-term toxicity test was conducted to examine the biosafety of CeO_2_@RSV (1 mg/kg). Then, HE staining showed no histological variation between the treatment groups and the control group in the heart, liver, spleen, lung, kidney and ovary (Additional file 1: Fig. S13). As shown in Additional file 1: Fig. S14A, B, the PCOS model mice were intravenously injected with indocyanine green (ICG)-labelled CeO_2_@RSV NPs, and the abdomen was enriched with abundant nanoparticles. Furthermore, we witnessed an obvious accumulation of NPs in ovarian tissue, among which the CeO_2_@RSV group displayed prominent bioavailability.

PCOS is known for its chronic inflammation status, and macrophage infiltration is the main pathological event that occurs in the ovaries of PCOS patients [45]. The ELISA results showed that the level of the proinflammatory cytokine IL1-β was decreased in all NPs-treated mice, and the level of the anti-inflammatory cytokine IL-10 was significantly increased in the CeO_2_@RSV group compared with the PCOS model mice (Fig. 4B), suggesting that inflammation was suppressed by NPs treatment and that CeO_2_@RSV exhibited the greatest effect. RT‒PCR analysis of the ovarian tissues also showed the same results (Fig. 4C). Similarly, the M1/M2 ratio in the spleen of CeO_2_@RSV-treated mice was greatly decreased (Fig. 4D), indicating that CeO_2_@RSV successfully altered the macrophage polarization state. Moreover, the apoptosis of ovarian GCs in PCOS mice was further investigated. As shown in Fig. 4E, the TUNEL assay showed that NPs treatment was able to suppress the apoptosis of ovarian GCs in PCOS mice, among which the CeO_2_@RSV group contributed the most efficiently. Similarly, western blot analysis confirmed a consistent trend (Fig. 4F). Overall, these results suggested that CeO_2_@RSV could effectively repolarize M1 macrophages into the M2 phenotype, alleviate the inflammatory response, and ameliorate GC apoptosis in the ovarian tissues of PCOS mice.

### Effects of CeO_2_@RSV on ameliorating ovarian function and endocrine disorders in PCOS mice

**Fig. 5:**
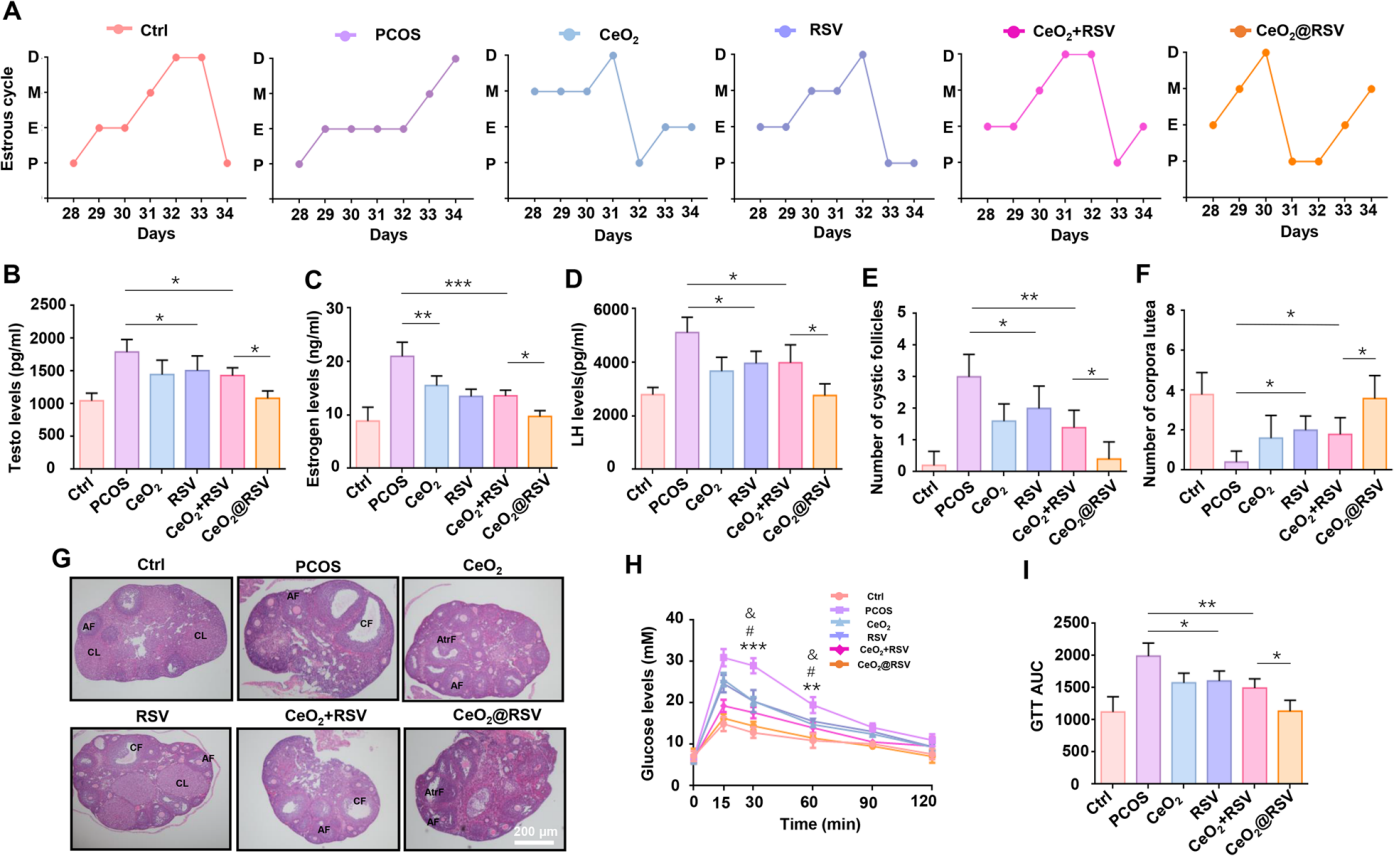
Effects of CeO_2_@RSV on ameliorating ovarian function and endocrine disorders in PCOS mice. **A** Cytological assessment of vaginal smears after inducing PCOS and treatment with NPs. **B**–**D** Serum levels of T, E_2_ and LH in the indicated groups. **E** The number of cystic follicles in the ovaries of the mice in the indicated groups. **F** The number of corpora lutea in the ovaries of the mice in the indicated groups. n = 5, *P < 0.05, **P < 0.01, ***P < 0.001. **G** Representative HE staining of ovarian sections from the experimental mice. Scale bar: 200 µm. **H**, **I** Glucose tolerance tests and AUCs of the experimental mice. n = 5, *P < 0.05, **P < 0.01, ***P < 0.001 vs. the CeO_2_ group; ^#^*P* < 0.05, ^##^*P* < 0.01 vs. the RSV group. ^&^*P* < 0.05 vs. the CeO_2_ + RSV group

Reportedly, ovarian dysfunction including endocrine, follicular growth and ovulation disorders, is an important characteristic of PCOS [46]. Given the excellent anti-inflammatory activity of CeO_2_@RSV, we then assessed its effects on ovarian function and endocrine disorders. As indicated in Fig. 5A, PCOS mice exhibited abnormal estrous cycles, and this phenomenon was attenuated by NPs treatment. Examination of hormones showed that the serum levels of estradiol (E_2_), testosterone (T) and luteinizing hormone (LH) were significantly higher in the PCOS group and subsequently decreased in all experimental groups, especially those treated with CeO_2_@RSV (Fig. 5B–D). In addition, the PCOS group showed the typical change in ovarian morphology, with a considerable number of cystic follicles (CF) and a relatively diminished number of corpora lutea (CL), and administration of NPs reversed these effects (Fig. 5E–G). According to the GTT results, PCOS mice exhibited impaired glucose tolerance, while treatment with NPs significantly suppressed glucose levels and the GTT area under the curve (AUC), and the CeO_2_@RSV group alleviated the levels most efficiently (Fig. 5H, I). These data suggested that CeO_2_@RSV treatment ameliorated endocrine disorders and abnormal ovarian morphology and restored impaired glucose metabolism in DHEA-induced PCOS mice.

### Anti-inflammatory mechanism of CeO_2_@RSV evaluated by transcriptome

**Fig. 6:**
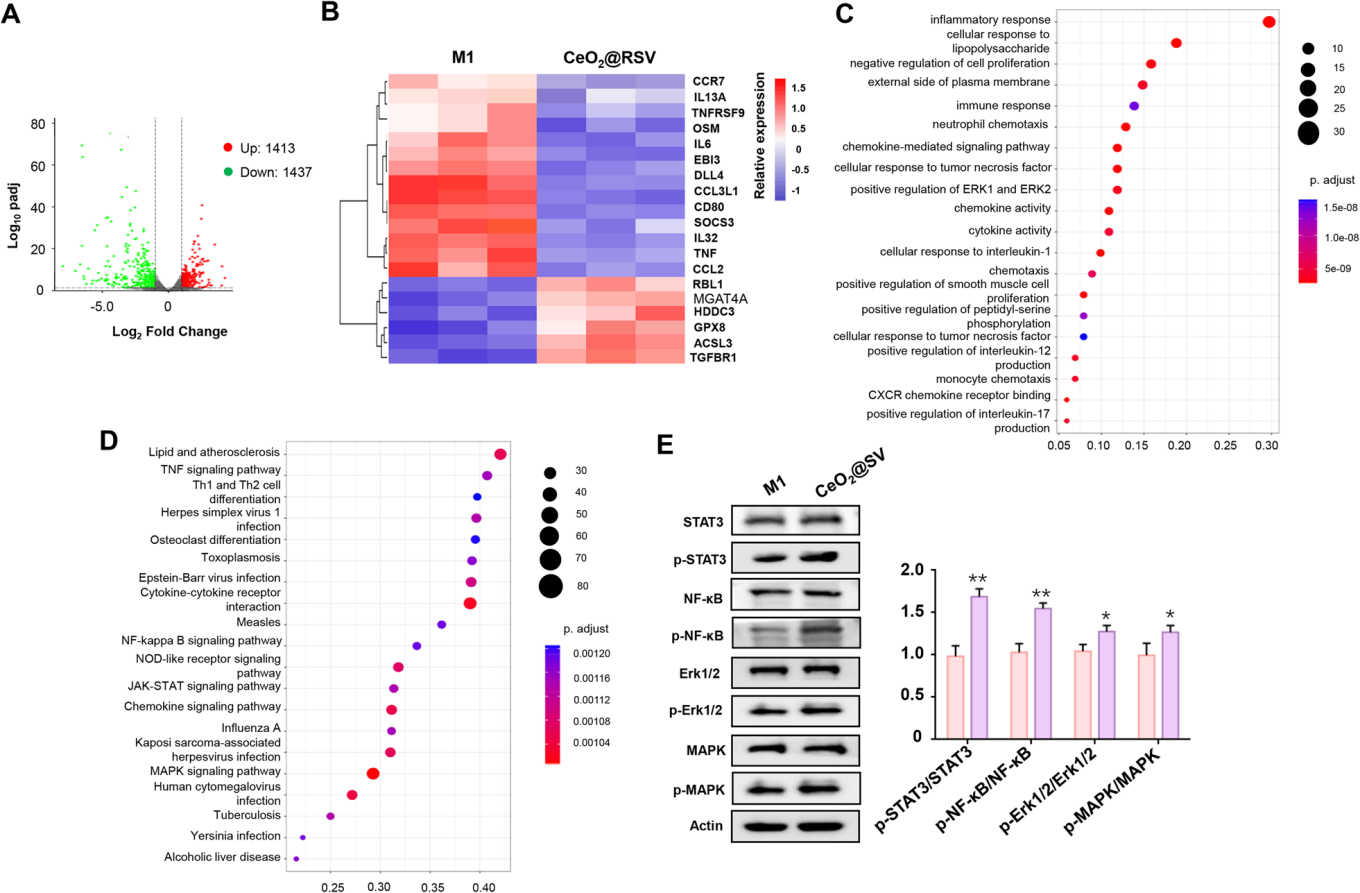
Anti-inflammatory mechanism of CeO_2_@RSV evaluated by transcriptome. **A** Volcano plots showing the genes regulated by treatment with CeO_2_@RSV. **B** Clustered heatmap of representative inflammation-related genes (fold change ≥ 2.0 and p < 0.05). **C** Bar plot of biological processes of differentially expressed genes between the M1 and CeO_2_@RSV groups. **D** KEGG pathway enrichment analysis of the differentially expressed genes between the M1 and CeO_2_@RSV groups. **E** The protein expression of STAT3/p-STAT3, NF-κB/p-NF-κB, Erk1/2/p-Erk1/2, and MAPK/p-MAPK was detected by western blotting. n = 3, *P < 0.05, **P < 0.01, ***P < 0.001 vs. the M1 group

To gain further insight into the anti-inflammatory mechanism of CeO_2_@RSV, RNA sequencing was performed in M1 macrophages treated with CeO_2_@RSV. Volcano plots demonstrated that the genes were significantly different after treatment with CeO_2_@RSV (Fig. 6A). As illustrated in Fig. 6B, the clustered heatmap shows that treatment with CeO_2_@RSV had a suppressive effect on various proinflammatory genes, such as IL13A, TNF, IL-6, CCL2 and IL32. Notably, the expression of anti-inflammatory and antioxidative genes, including GPX8 and TGFBR1, was distinctively upregulated after CeO_2_@RSV treatment compared to that in the M1 macrophage group. GO enrichment analysis of the differentially expressed genes demonstrated significant changes in immune response, cytokine activity, and chemokine activity (Fig. 6C). KEGG analysis identified significantly enriched pathways, and the TNF signaling pathway, cytokine‒cytokine receptor interaction, NF-κB signaling pathway, NOD-like receptor signaling pathway, JAK-STAT signaling pathway, and MAPK signaling pathway were strongly associated with the anti-inflammatory effects of CeO_2_@RSV (Fig. 6D). To further investigate the specific pathway after CeO_2_@RSV treatment, western blot analysis was conducted and the results revealed that the phosphorylation of STAT, NF-κB, Erk1/2 and MAPK was activated to varying degrees. Among them, p-STAT3/STAT3 was the most significant which suggested that the JAK-STAT signaling pathway played an important role.

### The JAK-STAT signaling pathway is responsive to the treatment of CeO_2_@RSV during PCOS

**Fig. 7:**
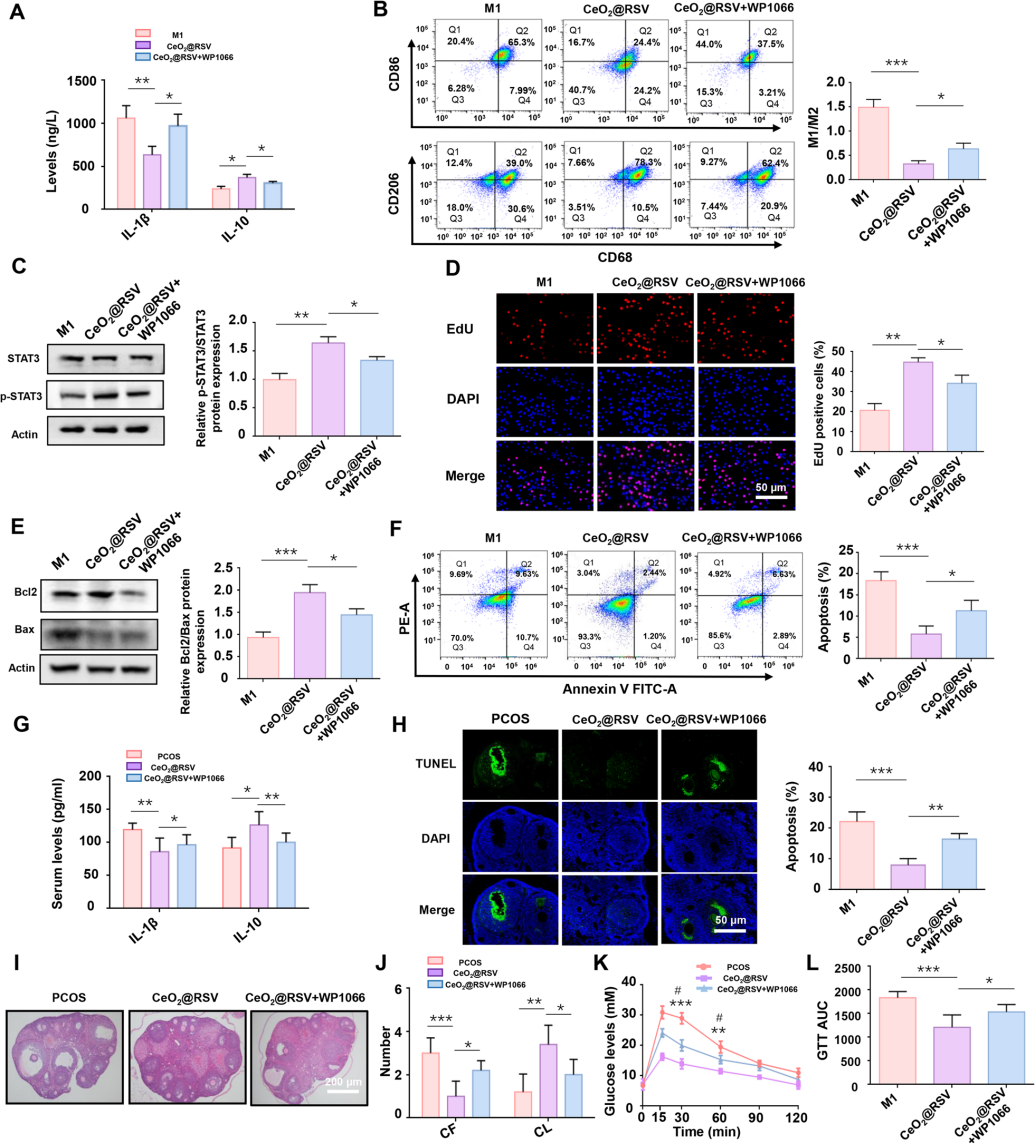
The JAK-STAT signaling pathway is responsive to the treatment of CeO_2_@RSV during PCOS. **A** ELISA results of IL-1β and IL-10 levels in the supernatants of CeO_2_@RSV-treated M1 macrophages incubated with or without WP1066. **B** Flow cytometry analysis of specific markers of M1 (CD86) and M2 (CD206) macrophages in different experimental treatment groups. **C** The protein expression of STAT3 and p-STAT3 in the three groups. **D** Representative images and quantification of EdU (+) in KGN cells. **E** Western blot analysis of Bcl2/Bax in KGN cells. **F** Apoptosis of KGN cells in each group was detected by flow cytometry. **G** ELISA results of IL1-β and IL-10 levels in the serum of each group. **H** A TUNEL assay was used to detect apoptosis in ovarian tissues in vivo. **I** Representative HE staining of ovarian sections from the experimental mice. **J** The number of CFs and CLs in the ovaries of the mice in each group. *P < 0.05, **P < 0.01, ***P < 0.001. **K**, **L** Glucose tolerance tests and AUCs of the indicated groups. n = 5, *P < 0.05, **P < 0.01, ***P < 0.001 vs. the PCOS group; ^#^*P* < 0.05, ^##^*P* < 0.01 vs. the CeO_2_@RSV group

Given the potent efficacy of CeO_2_@RSV and the transcriptome analysis, relevant mechanisms of CeO_2_@RSV-mediated macrophage polarization and the treatment of PCOS were explored, and relevant in vivo and in vitro experiments were conducted for further verification. In cell experiments, we found that a specific inhibitor of JAK (WP1066) reversed the effect of CeO_2_@RSV on macrophage polarization, with elevated levels of M1 phenotype markers and depressed levels of M2 phenotype markers (Fig. 7A, B). Additionally, western blot analysis showed that after inhibiting JAK, STAT3 phosphorylation decreased (Fig. 7C). At the same time, WP1066 inhibited the increased Bcl2/Bax expression and promoted the apoptosis of KGN cells after coculture with M1 macrophages (Fig. 7D–F). In animal experiments, PCOS mice were then administered CeO_2_@RSV + WP1066, and ELISA, western blotting, TUNEL and HE staining were performed. The results indicated that after inhibiting JAK, the serum level of IL-1β was enhanced, but IL-10 was reduced (Fig. 7G). The TUNEL assay showed that the apoptosis of ovarian tissues was increased when WP1066 was administered (Fig. 7H). Furthermore, WP1066 treatment deteriorated ovarian function, which was improved by CeO_2_@RSV, with an increased number of CFs, a decreased number of CLs and deteriorated glucose tolerance (Fig. 7I–L). All of these results suggested that the JAK-STAT signaling pathway is a key signaling pathway in CeO_2_@RSV therapy for PCOS, which involves changes in the immune microenvironment, ovarian function, and glucose metabolism.

## Discussion

PCOS is a disorder with reproductive, endocrine and metabolic irregularities that poses enormous fertility challenges for women of reproductive age [47]. It is known that anti-inflammation is an important strategy in the treatment of PCOS syndromes [48, 49]. Traditional agents for PCOS therapy (such as metformin and oral contraceptives) mainly alleviate a series of complications, have poor stability and cause severe side effects [50, 51], and there is a need to develop novel treatment options. Macrophages are key mediators of the immune response in the ovarian immune microenvironment due to their considerable plasticity and versatile roles in response to external stimuli [52]. It has been well documented that aberrant macrophage polarization is critical for the maintenance of proinflammatory phenotypes during PCOS and accelerates the injury of ovarian tissues [53]. Therefore, rebalancing proinflammatory microenvironment agents would be beneficial for precise PCOS therapy.

Recently, CeO_2,_ with potent anti-inflammatory and antioxidant effects, has attracted considerable attention and protects against numerous inflammation-related diseases, such as diabetic wounds, acute kidney injury and ischemic stroke [54, 55]. However, no study has focused on the therapeutic effect of nanomaterials on PCOS, and current CeO_2_ nanoparticles have been limited by their anti-inflammatory effects on the M2 phenotype. In the present study, we characterized a novel nanoparticle, CeO_2_@RSV, which was synthesized via chemical bonding of CeO_2_ with traditional anti-inflammatory RSV, thus exhibiting satisfactory effects in the treatment of PCOS. Compared with traditional anti-inflammatory or natural enzymes, the chemically bonded nanozyme CeO_2_@RSV shows superior capability in eradicating the inflammatory response, scavenging excessive ROS and simultaneously exhibiting excellent biocompatibility. Our results demonstrated that CeO_2_@RSV possessed prominent SOD- and CAT-mimetic activities and has tremendous potential to protect cells from a variety of types of damage. Macrophages are the key regulators of GC proliferation, follicular development and ovulation [14], and the balance of the M1/M2 ratio determines the pathophysiology of PCOS [16]. After being taken up by macrophages, CeO_2_@RSV can efficiently manipulate the repolarization of macrophages, thereby reducing the proinflammatory M1 phenotype and enhancing the anti-inflammatory M2 phenotype. In addition, increasing evidence emphasizes the great potential of crosstalk between macrophages and granulosa cells in the physiological functions of ovaries [14]. Upon inflammatory stimulation, macrophage recruitment and subsequent proinflammatory cytokine release induce GC apoptosis and follicular atresia [56]. As expected, our results demonstrated that CeO_2_@RSV-mediated macrophage transformation promoted the proliferation and inhibited the apoptosis of ovarian granulosa cells. Moreover, our results showed that CeO_2_@RSV treatment ameliorated endocrine disorders and abnormal ovarian morphology, and restored impaired glucose metabolism in DHEA-induced PCOS mice. Therefore, all the results suggest that alterations in the local microenvironment mediated by activated M1/M2 subtypes regulate the function of the ovary during PCOS.

Within the ovary, macrophage activities are involved in various aspects of ovarian function in PCOS pathogenesis [57]. ELISA and flow cytometry analysis revealed that treatment with CeO_2_@RSV dramatically decreased the number of proinflammatory macrophages in PCOS mice, which may contribute to the dampened proinflammatory microenvironment. RNA-seq data and further experiments indicated that several signaling pathways were related to the role of CeO_2_@RSV in macrophages, including the JAK-STAT signaling pathway, NF-κB signaling pathway and MAPK signaling pathway, of which the JAK-STAT signaling pathway exhibited the most marked effects. The enrichment analysis revealed that the immune response, cytokine activity, and chemokine activity are vital changes after CeO_2_@RSV treatment, which are significantly correlated with the improvement of PCOS. At the same time, the JAK/STAT pathway emerges as a major regulator of macrophage polarization that characteristically influences the production of proinflammatory cytokines, which are vital for the development of inflammatory diseases [58, 59]. Further explorations confirmed that the therapeutic abilities of CeO_2_@RSV could be inhibited by inhibitors of the JAK/STAT pathway in vitro and in vivo.

## Conclusion

Nanoparticles have emerged as a promising approach that possesses high biocompatibility and drug delivery capabilities. The integration of traditional anti-inflammatory drugs and various functions of synthetic materials endow nanoparticles with widespread implications [60]. In our study, RSV was loaded with CeO_2_ via chemical bonding to obtain the anti-inflammatory nanoparticle CeO_2_@RSV. CeO_2_@RSV nanoparticles preferentially suppress inflammation response-mediated injury and alleviate endocrine dysfunction, thereby reversing the pathological changes that occur during the development of PCOS. Overall, our study may elucidate the treatment and management of PCOS by using nanoparticles as potential therapeutics.

### Supplementary Information

**Additional file 1: Table S1.** Baseline characteristics of the study population (n = 10). **Table S2.** Primer sequences for RT‐PCR. **Table S3.** EE and LE of different concentration of RSV onto CeO_2_@APTES. **Figure S1.** The schematic graph of the chemical bonding between resveratrol and APTES. **Figure S2.** Encapsulation efficiency and loading efficiency of CeO_2_@APTES NPs with different RSV concentration. **Figure S3.** The diameters of the NPs were determined by TEM. **Figure S4.** Representative EDX mapping images of Ce, N, and C elements in CeO_2_@RSV nanoparticles. Scale bar: 100 nm. **Figure S5.** XRD measurement of CeO_2_, CeO_2_@APTES and CeO_2_@RSV. **Figure S6.** XPS analysis of CeO_2_ and CeO_2_@RSV nanoparticles. **Figure S7.** ABTS^+^ scavenging effects of different samples. **Figure S8.** The stability of different samples. Zeta potentials (A) and average size (B) of prepared NPs. **Figure S9.** Cell viability of THP1 cells in CCK-8 assay incubated with CeO_2_@RSV NPs at a concentration of 10, 50 and 100 µg/mL for 24, 48 and 72 h. Data are shown as the mean ± SD. n = 3, *P < 0.05, **P < 0.01 vs. the control group. **Figure S10.** CeO_2_@RSV could drive macrophage polarization and reduce inflammatory response. (A). The diagram described the effect of CeO_2_@RSA on macrophage polarization. (B). The expression levels of polarization markers in these groups were determined by RT-PCR (C) western blot (D) and flow cytometry analyses (E) with different experimental treatment. n = 3, *P < 0.05, **P < 0.01, ***P < 0.001. **Figure S11.** CeO_2_@RSV treatment attenuated oxidative stress. Levels of SOD (A) and MDA (B) in M1 macrophages from each group 48 h after NP treatment (n = 3). *P < 0.05, **P < 0.01, **P < 0.001. **Figure S12.** CeO_2_@RSV-induced macrophage polarization promotes proliferation and inhibits apoptosis in granulosa cells. (A). RT-PCR analysis of Bcl2/Bax in different groups. (B). Western blot analysis of Bcl2/Bax in different groups. (C). Apoptosis of granulosa cells in each group was detected by flow cytometry. n = 3, *P < 0.05, **P < 0.01. **Figure S13.** Evaluation on the biocompatibility of the CeO_2_@RSV from C57BL/6 mice. Mice treated with PBS were defined as a control group. n = 5, scale bar: 50 µm. **Figure S14.** (A). In vivo fluorescence imaging of mice and Ex vivo fluorescence images of organs received intravenous injection of CeO_2_@RSV NPs. (B). The fluorescence images were detected in the ovary tissue injected with CeO_2_, CeO_2_ + RSV and CeO_2_@RSV NPs.

## Data Availability

All generated data was included in the present study.

## Abbreviations

PCOS: Polycystic ovary syndrome
DHEA: Dehydroepiandrosterone
ROS: Reactive oxygen species
GCs: Granulosa cells
NPs: Nanoparticles
RSV: Resveratrol
APTES: 3-Aminopropyltriethoxysilane
CeO_2_: Nanoceria
MDA: Malondialdehyde
SOD: Superoxide dismutase
CAT: Catalase
OGTT: Oral glucose tolerance test
XPS: X-ray photoelectron spectroscopy
E_2_: Estradiol
T: Testosterone
LH: Luteinizing hormone
CF: Cystic follicles
CL: Corpora lutea
