# Supplementary material for: Nanocomposites based on nanoceria regulate the immune microenvironment for the treatment of polycystic ovary syndrome
Source: J Nanobiotechnology. 2023 Nov 7;21:412. doi: 10.1186/s12951-023-02182-w (PMC10631133; doi:10.1186/s12951-023-02182-w)
Supplement: Supplementary file 1 — Additional file 1: Table S1. Baseline characteristics of the study population (n = 10). Table S2. Primer sequences for RT‐PCR. Table S3. EE and LE of different concentration of RSV onto CeO2@APTES. Figure S1. The schematic graph of the chemical bonding between resveratrol and APTES. Figure S2. Encapsulation efficiency and loading efficiency of CeO2@APTES NPs with different RSV concentration. Figure S3. The diameters of the NPs were determined by TEM. Figure S4. Representative EDX mapping images of Ce, N, and C elements in CeO2@RSV nanoparticles. Scale bar: 100 nm. Figure S5. XRD measurement of CeO2, CeO2@APTES and CeO2@RSV. Figure S6. XPS analysis of CeO2 and CeO2@RSV nanoparticles. Figure S7. ABTS+ scavenging effects of different samples. Figure S8. The stability of different samples. Zeta potentials (A) and average size (B) of prepared NPs. Figure S9. Cell viability of THP1 cells in CCK-8 assay incubated with CeO2@RSV NPs at a concentration of 10, 50 and 100 µg/mL for 24, 48 and 72 h. Data are shown as the mean ± SD. n = 3, *P < 0.05, **P < 0.01 vs. the control group. Figure S10. CeO2@RSV could drive macrophage polarization and reduce inflammatory response. (A). The diagram described the effect of CeO2@RSA on macrophage polarization. (B). The expression levels of polarization markers in these groups were determined by RT-PCR (C) western blot (D) and flow cytometry analyses (E) with different experimental treatment. n = 3, *P < 0.05, **P < 0.01, ***P < 0.001. Figure S11. CeO2@RSV treatment attenuated oxidative stress. Levels of SOD (A) and MDA (B) in M1 macrophages from each group 48 h after NP treatment (n = 3). *P < 0.05, **P < 0.01, **P < 0.001. Figure S12. CeO2@RSV-induced macrophage polarization promotes proliferation and inhibits apoptosis in granulosa cells. (A). RT-PCR analysis of Bcl2/Bax in different groups. (B). Western blot analysis of Bcl2/Bax in different groups. (C). Apoptosis of granulosa cells in each group was detected by flow cytometry. n = 3, *P [file 12951_2023_2182_MOESM1_ESM.docx]

**Nanocomposites based on nanoceria through regulation of the immune microenvironment for the treatment of polycystic ovary syndrome**

Sisi Yan^1^+ | Zhipeng Gao^2^+ | Jinli Ding^1^+ | Suming Chen^3^ | Zehao Wang^1^ | Wenyi Jin^1^ | Bing Qu^4^ | Yi Zhang^1^ | Lian Yang^1^ | Duan Ying Guo^5^*| Tailang Yin^1^* | Yanbing Yang^2^* | Yan Zhang^6^* | Jing Yang^1^*

1. Reproductive Medical Center, Renmin Hospital of Wuhan University and Hubei Clinic Research center for Assisted Reproductive Technology and Embryonic Development, Wuhan, China
2. College of Chemistry and Molecular Sciences, Key Laboratory of Biomedical Polymers of Ministry of Education, Wuhan University, Wuhan, China
3. The Institute for Advanced Studies, Wuhan University, Wuhan, China
4. Department of General Surgery, Renmin Hospital of Wuhan University, Wuhan, China
5. Longgang District People's Hospital of Shenzhen
6. Department of Clinical Laboratory, Renmin Hospital of Wuhan University, WuHan, HuBei, China

***Correspondence***

Jing Yang, Reproductive Medical Center, Renmin Hospital of Wuhan University and Hubei Clinic Research Center for Assisted Reproductive Technology and Embryonic Development, WuHan 430060, China.

E-mail: [dryangjing@whu.edu.cn](mailto:dryangjing@whu.edu.cn).

Yan Zhang, Department of Clinical Laboratory, Renmin Hospital of Wuhan University, WuHan, HuBei, China.

E-mail: peneyyan@mail.ustc.edu.cn.

Yanbing Yang, College of Chemistry and Molecular Sciences, Key Laboratory of Biomedical Polymers of Ministry of Education, Wuhan University, Wuhan 430072, P. R. China.

E-mail: [yangyanbing@whu.edu.cn](mailto:yangyanbing@whu.edu.cn).

Tailang Yin, Reproductive Medical Center, Renmin Hospital of Wuhan University and Hubei Clinic Research Center for Assisted Reproductive Technology and Embryonic Development, Wuhan 430060, China.

E-mail: [reproductive@whu.edu.cn](mailto:reproductive@whu.edu.cn).

Duan Ying Guo, Longgang District People's Hospital of Shenzhen, Shenzhen, China.

E-mail: guo.duanying@163.com

* Sisi Yan, Zhipeng Gao and Jinli Ding contributed equally to this work.

1. **Additional tables and figures**

**Table S1.** Baseline characteristics of the study population (n=10).

|  | PCOS (n=10) |
| --- | --- |
| Age (y) | 30.91±1.94 |
| BMI (kg/m^2^) | 23.02 ± 1.76 |
| Serum AMH (ng/mL) | 8.87 ± 2.53 |
| FSH (mIU/mL) | 7.01 ± 1.75 |
| T(ng/dL) | 43.26 ± 6.14 |
| LH (mIU/mL) | 12.02 ± 3.32 |
| E_2_ (pmol/L) | 49.58 ± 5.73 |

BMI, body mass index; AMH, Anti-mullerian hormone; FSH, follicle stimulating hormone; T, testosterone; LH, luteinizing hormone; E_2_, estradiol. Data are presented as mean ± SD.

[**Table S2**](#ec0005)**.** Primer sequences for RT‐PCR.

| Gene | Primer Sequence (5’ to 3’) |
| --- | --- |
| H-Actin | F: AACCGCGAGAAGATGACCCAG  R: GTCACCGGAGTCCATCACGAT |
| H-IL-6 | F: ACTCACCTCTTCAGAACGAATTG  R: CCATCTTTGGAAGGTTCAGGTTG |
| TNF-α | F: TCTCGAACCCCGAGTGACAA  R: TGAAGAGGACCTGGGAGTAG |
| H-IL-1β | F: TGAACTGAAAGCTCTCCACC  R: CTGATGTACCAGTTGGGGAA |
| H-iNOS | F: TTCAGTATCACAACCTCAGCAAG  R: TGGACCTGCAAGTTAAAATCCC |
| H-CD206 | F: GGGTTGCTATCACTCTCTATGC  R: TTTCTTGTCTGTTGCCGTAGTT |
| H- IL-10 | F: GACTTTAAGGGTTACCTGGGTTG  R: TCACATGCGCCTTGATGTCTG |
| H-Arg1 | F: GTGGAAACTTGCATGGACAAC  R: AATCCTGGCACATCGGGAATC |
| H-Bcl2 | F: GGATGCCTTTGTGGAACTGT  R: CACTTGTGGCTCAGATAGGC |
| H-Bax | F: CACTGAAGCGACTGATGTC  R: TCAGCCCATCTTCTTCCA |
| M- IL-6 | F: CTGCAAGAGACTTCCATCCAG  R: AGTGGTATAGACAGGTCTGTTGG |
| M-iNOS | F: CCAAGCCCTCACCTACTTCC  R: CTCTGAGGGCTGACACAAGG |
| M-IL-10 | F: GCTCTTACTGACTGGCATGAG  R: CGCAGCTCTAGGAGCATGTG |

**Table S3.** EE and LE of different concentration of RSV onto CeO_2_@APTES.

| Amount of RSV (mg) | EE (%) | LE (%) |
| --- | --- | --- |
| 5 | 71.83 ± 2.8 | 3.42 ± 0.45 |
| 10 | 67.92 ± 2.32 | 6.13 ± 1.07 |
| 15 | 63.4 ± 3.03 | 7.81 ± 1.16 |


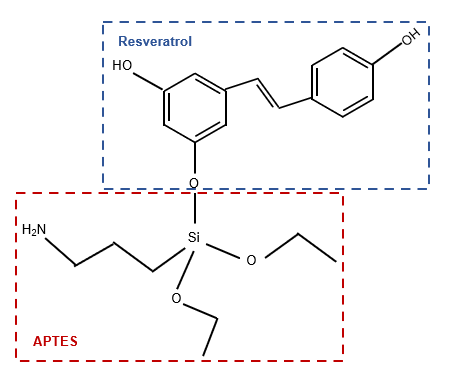


**Figure S1.** The schematic graph of the chemical bonding between resveratrol and APTES


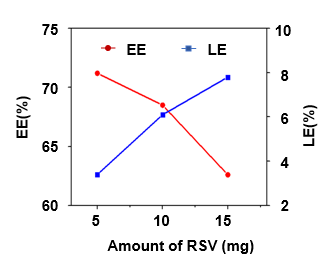


**Figure S2.** Encapsulation efficiency and loading efficiency of CeO_2_@APTES NPs with different RSV concentration.


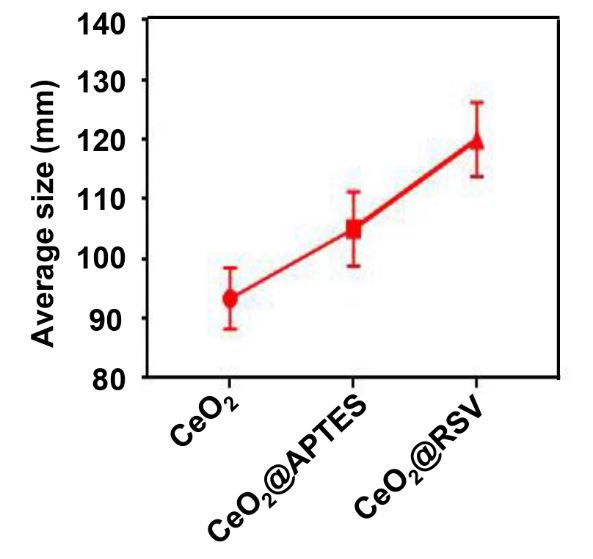


**Figure S3.** The diameters of the NPs were determined by TEM.


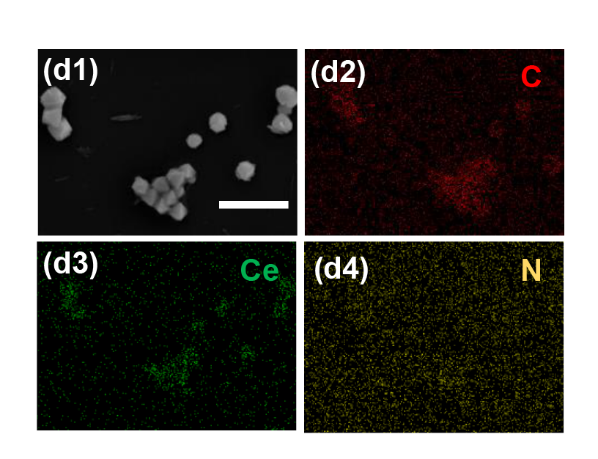


**Figure S4.** Representative EDX mapping images of Ce, N, and C elements in CeO_2_@RSV nanoparticles. Scale bar: 100 nm.


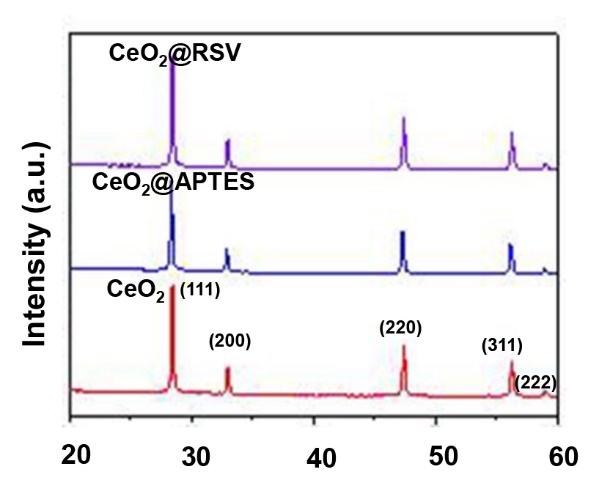


**Figure S5.** XRD measurement of CeO_2_, CeO_2_@APTES and CeO_2_@RSV.


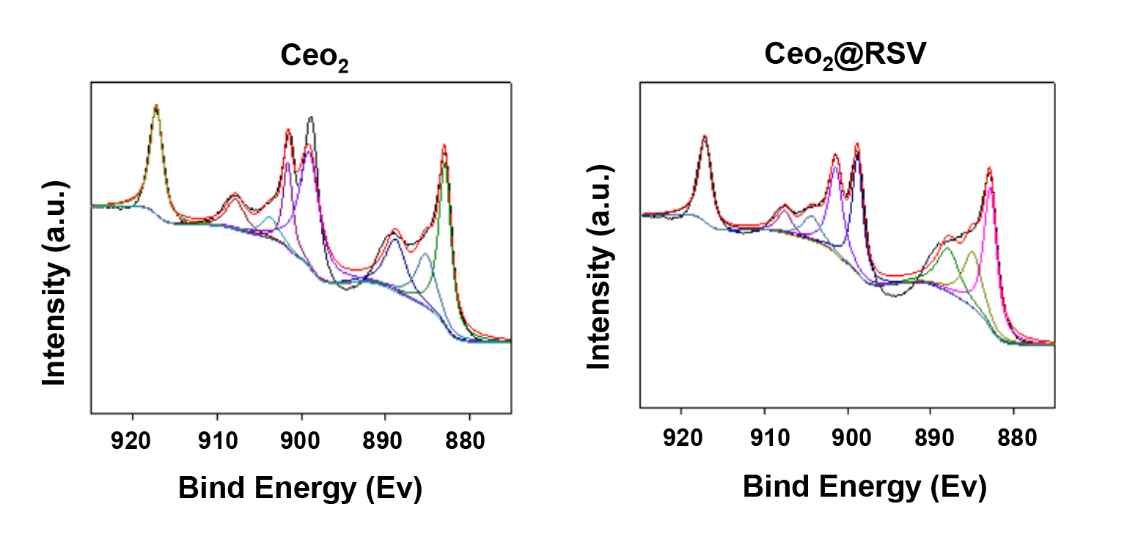


**Figure S6.** XPS analysis of CeO_2_ and CeO_2_@RSV nanoparticles.


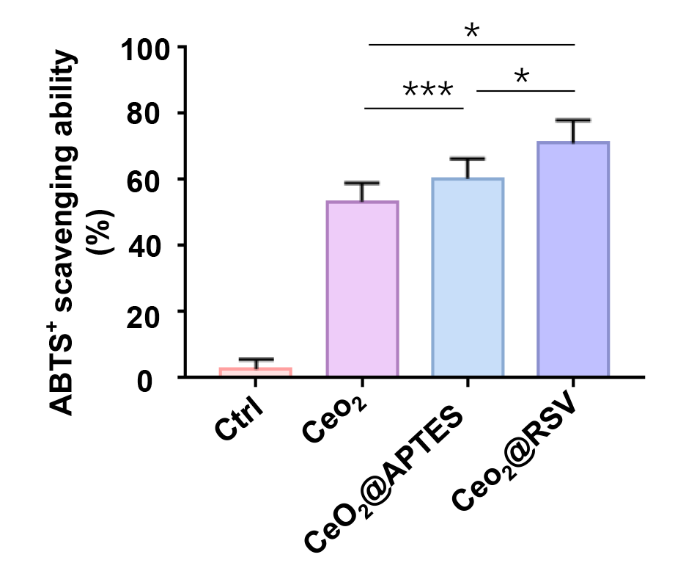


**Figure S7.** ABTS^+^ scavenging effects of different samples.


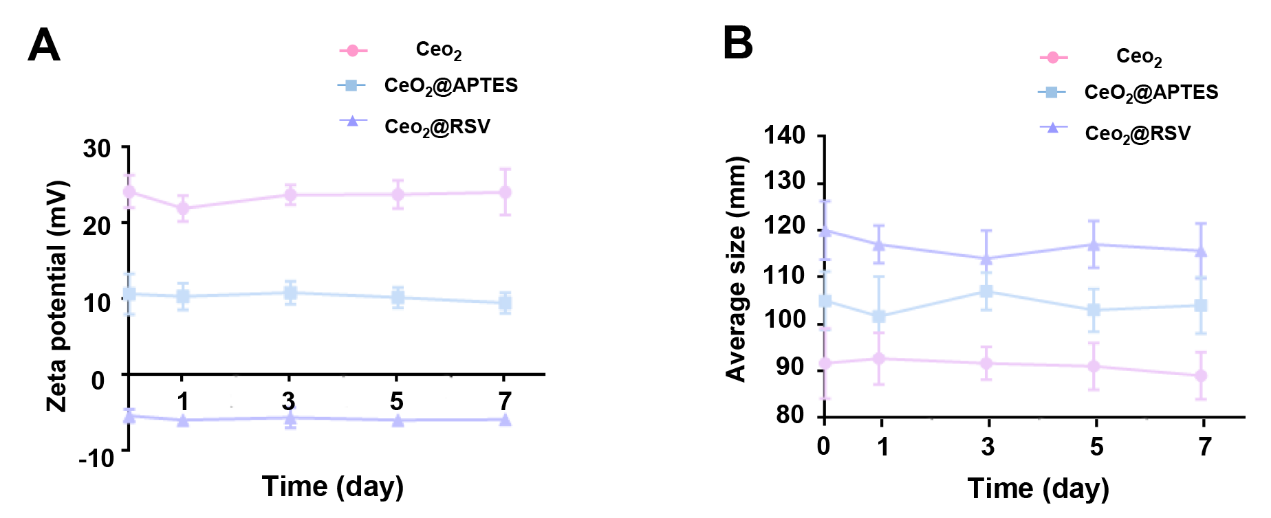


**Figure S8.** The stability of different samples. Zeta potentials (A) and average size (B) of prepared NPs.


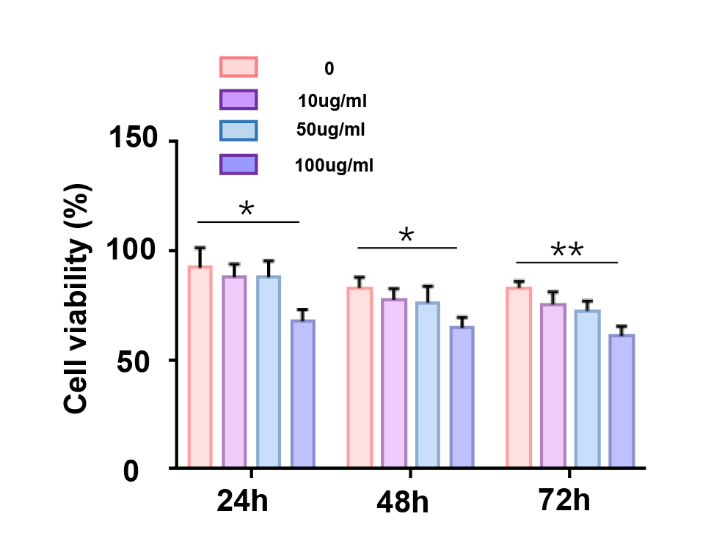


**Figure S9.** Cell viability of THP1 cells in CCK-8 assay incubated with CeO_2_@RSV NPs at a concentration of 10, 50 and 100 ug/ml for 24, 48 and 72h. Data are shown as the mean ± SD. n = 3, ^*^*P*<0.05, ^**^*P*<0.01 vs. the control group.


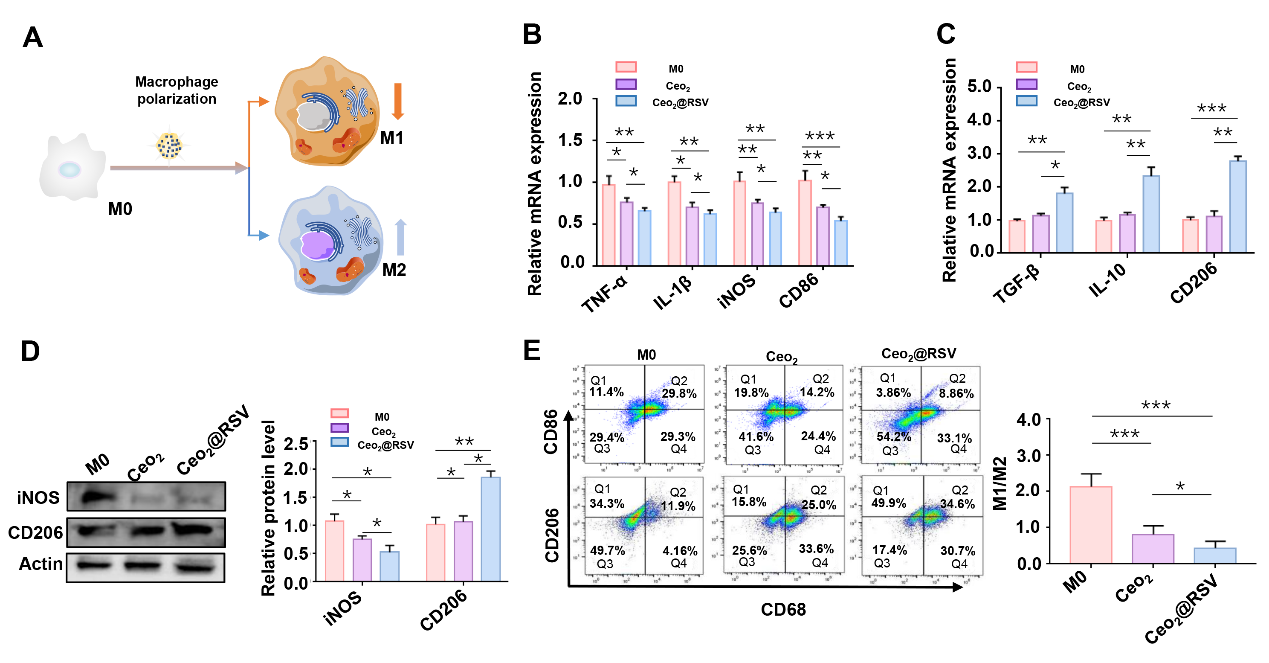
**Figure S10.** CeO_2_@RSV could drive macrophage polarization and reduce inflammatory response. (A). The diagram described the effect of CeO_2_@RSA on macrophage polarization. (B). The expression levels of polarization markers in these groups were determined by RT-PCR (C) western blot (D) and flow cytometry analyses (E) with different experimental treatment.  n = 3, ^*^*P*<0.05, ^**^*P*<0.01, ^***^*P*<0.001.


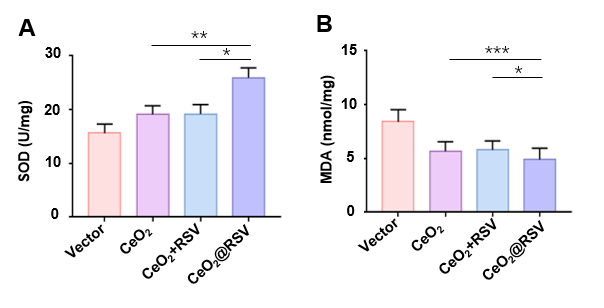


**Figure S11.** CeO_2_@RSV treatment attenuated oxidative stress. Levels of SOD (A) and MDA (B) in M1 macrophages from each group 48 h after NP treatment (n = 3). **P*<0.05, ***P*<0.01, ***P*<0.001.


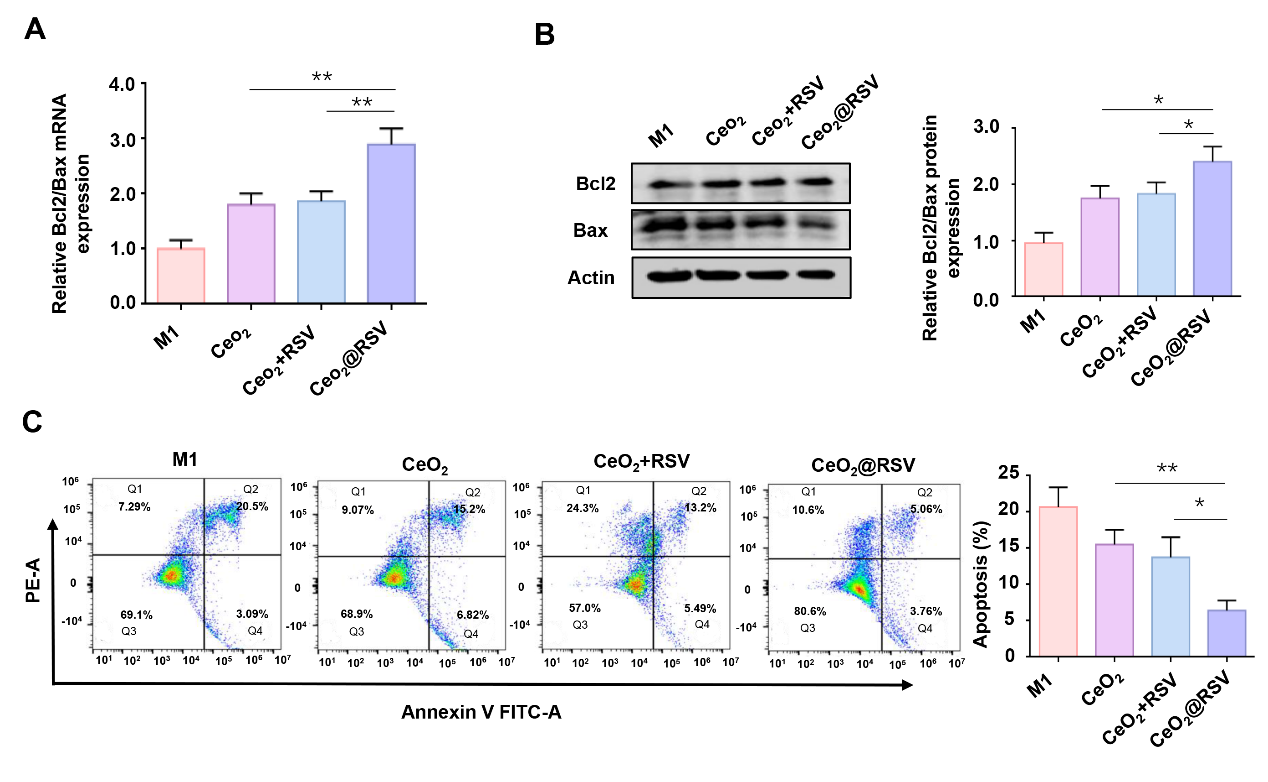


**Figure S12. CeO_2_@RSV-induced macrophage polarization promotes proliferation and inhibits apoptosis in granulosa cells.** (A). RT-PCR analysis of Bcl2/Bax in different groups. (B). Western blot analysis of Bcl2/Bax in different groups. (C). Apoptosis of granulosa cells in each group was detected by flow cytometry. n = 3, ^*^*P*<0.05, ^**^*P*<0.01.


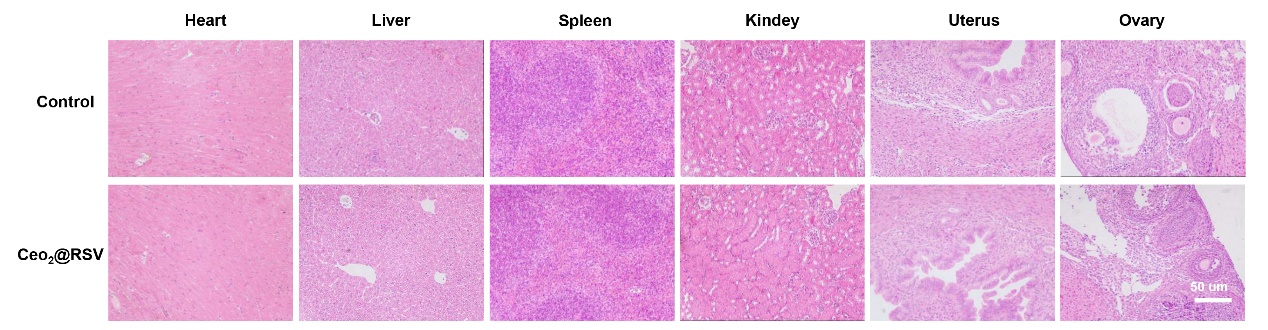


**Figure S13.** Evaluation on the biocompatibility of the CeO_2_@RSV from C57BL/6 mice. Mice treated with PBS were defined as a control group. n = 5, scale bar: 50 µm.


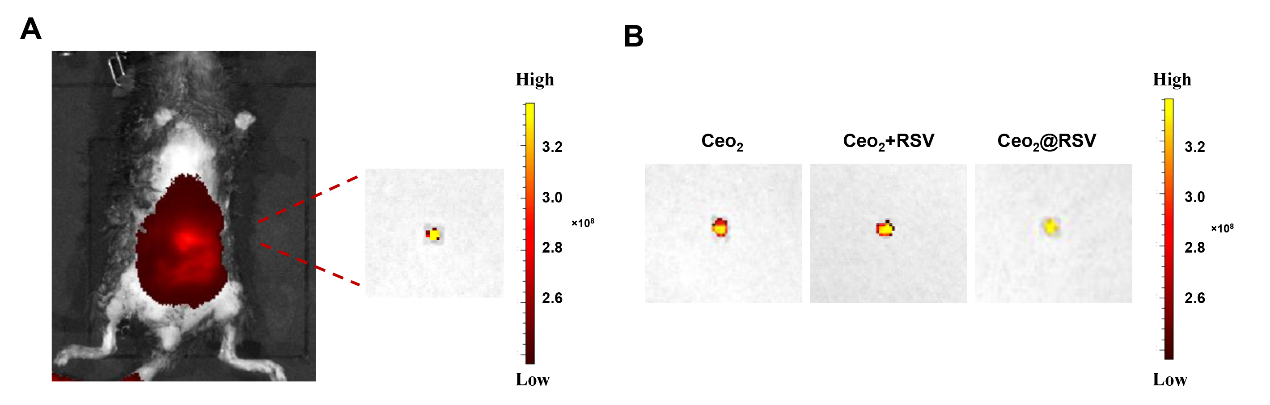


**Figure S14.** (A). *In vivo* fluorescence imaging of mice and Ex vivo fluorescence images of organs received intravenous injection of CeO_2_@RSV NPs. (B). The fluorescence images were detected in the ovary tissue injected with CeO_2_, CeO_2_+RSV and CeO_2_@RSV NPs.
